# π‑Conjugated Indole-Based 2D Perovskite Microcavities for Self-Powered Ultraviolet Photodetection

**DOI:** 10.1021/acs.chemmater.6c01061

**Published:** 2026-07-22

**Authors:** Meenakshi Pegu, Mukesh K. Thakur, Sudhir K. Saini, Aarya Prabhakaran, Gabriele Saleh, Mehmet Z. Akgul, Shence Zhang, Francesco De Boni, Sergio Marras, Simone Lauciello, Luca Goldoni, Matteo Lorenzoni, Serena De Negri, Pavlo Solokha, Roman Krahne, Liberato Manna

**Affiliations:** † Nanochemistry, 121451 Istituto Italiano di Tecnologia, Via Morego 30, Genova 16163, Italy; ‡ Photonic Nanomaterials, Istituto Italiano di Tecnologia, Via Morego 30, Genova 16163, Italy; § Optoelectronics Research Line, Istituto Italiano di Tecnologia, Via Morego 30, Genova 16163, Italy; ∥ Materials Characterization Facility, Istituto Italiano di Tecnologia, Via Morego 30, Genova 16163, Italy; ⊥ Electron Microscopy, Istituto Italiano di Tecnologia, Via Morego 30, Genova 16163, Italy; # Dipartimento di Chimica e Chimica Industriale, 9302 Università Degli Studi di Genova, Via Dodecaneso 31, Genova 16146, Italy; ¶ Department of Chemistry, Materials and Chemical Engineering, Politecnico di Milano, Piazza Leonardo da Vinci 32, Milano 20133, Italy

## Abstract

We report the synthesis and optoelectronic characterization of a two-dimensional layered halide perovskite (2DLP), TPA_2_PbBr_4_, incorporating tryptammonium (TPA) as a π-conjugated organic spacer molecule. TPA-Br, derived from indole-containing tryptamine, enables CH-π and NH-π interactions, enhancing structural rigidity and electronic coupling with the layered framework. TPA_2_PbBr_4_ microcrystals grown via a slow vapor diffusion method exhibit a highly oriented Ruddlesden–Popper-type structure, confirmed by powder and single-crystal X-ray diffraction (XRD) analyses. In situ temperature-dependent XRD confirms high thermal stability without phase transition from 183 to 433 K. Optical measurements reveal a sharp excitonic absorption peak near 398 nm and a narrow photoluminescence peak at 407 nm. Uniform spin-coated thin films display an excitonic double peak and pronounced Fabry–Pérot microcavity modes in their reflectance spectra, indicating strong optical confinement. The Fabry–Pérot microcavity sustained by the TPA_2_PbBr_4_ thin films facilitates such strong light-matter coupling, manifested by the formation of polariton modes. Planar photodetectors (PDs) with TPA_2_PbBr_4_ films exhibit stable ultraviolet photoresponse, low dark current, responsivity (*R*) of 1 mA/W, and detectivity (D*) of 2.2 × 10^10^ Jones. Vertically configured PDs based on TPA_2_PbBr_4_ thin films exploit their built-in electric field, enabling self-powered operation. The combination of strong excitonic features, microcavity-enhanced light absorption, and self-biased device operation highlights TPA_2_PbBr_4_ as a promising material for UV-selective PDs and low-power optoelectronic applications, benefiting from its strong excitonic features and π-conjugated organic spacer chemistry.

## Graphical abstract

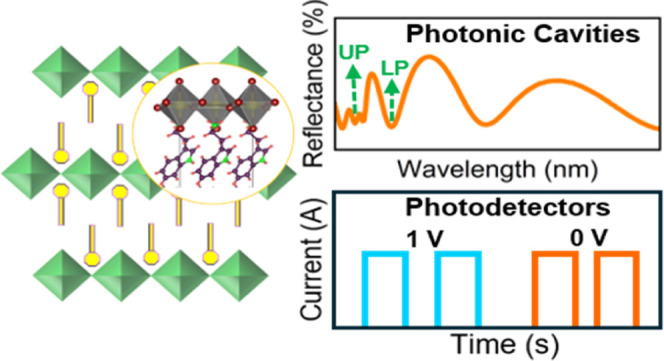

## 1 Introduction

Two-dimensional layered halide perovskites (2DLPs) have emerged as a promising class of semiconducting materials for optoelectronic applications due to their superior tunability, unique excitonic features, large absorption coefficients, quantum-well-like structures, and inherent stability compared to their three-dimensional (3D) analogues.^1–5^ 2D hybrid layered perovskites have the general formula of (R-NH_3_)_2_A_*n*–1_B_*n*_X_3*n*+1_, wherein long or short organic chain or spacer cations (R) isolate the inorganic perovskite slabs (with small organic or inorganic cations such as methylammonium MA^+^, formamidinium FA^+^, Cs^+^ as A-site cations; Pb^2+^, Sn^2+^, Ge^2+^ or Cu^2+^ as the B-site cations, and X as the halide anions, i.e., Cl^–^, Br^–^, or I^–^).^4,6–8^ The tunability of their optical and electronic properties through the choice of various organic chains or spacer cations makes them ideal candidates for applications in photovoltaics (PVs), light-emitting diodes (LEDs), and photodetectors (PDs).^9–15^

2DLPs are intensively explored for photodetection, as these materials provide the high absorption coefficients and charge generation efficiency required for narrowband and broadband PD devices.^16–18^ 2DLPs exhibit significantly improved resistance to moisture and thermal degradation, owing to the organic spacer layers that act as a barrier to moisture penetration and ion migration, which is advantageous for perovskite-based optoelectronic devices.^9,19^ Moreover, their strong excitonic absorption and sharp band-edge features are beneficial for narrowband and wavelength-selective photodetection.^20^ Recent developments in the field have focused on exploring new organic spacer cations to enhance the performance and stability of 2DLP-based PDs.^21–23^ Notably, short-chain alkylammonium and arylammonium halides as spacer cations have been widely explored.^2,24^ For instance, butylammonium (BA) and phenethylammonium (PEA) spacers have been investigated to produce thin films with controlled crystallinity and specific orientation, which is essential for charge transport in optoelectronic devices.^18,25^ Fluorinated aryl spacers have been employed to enhance the hydrophobicity and lower the density of defect states in thin films for device applications.^26,27^ All these strategies have led to significant improvements in device parameters such as responsivity, detectivity, photoresponse time, and signal-to-noise ratio.^28^ Another key role in achieving high-performance 2DLP PDs lies in the device architecture. Various architectures have been explored, including planar heterojunctions, interdigitated electrodes, and the vertical stacking of photodiodes.^29–31^ Furthermore, highly uniform 2DLP thin films can act as self-formed Fabry–Pérot microcavities, where refractive index contrast at the film interfaces induces internal reflections that generate resonant cavity modes.^32–34^ These cavity modes enhance strong light confinement, thereby facilitating more efficient absorption near the excitonic feature of the active layer. Under sufficiently strong coupling between the confined photons and the tightly bound excitons of the 2DLPs, the cavity mode and exciton resonance hybridize to form upper and lower polariton branches.^35,36^ Previous studies have demonstrated strong light-matter coupling in 2D perovskite single crystals, thick flakes, and mirror-assisted cavities (e.g., DBR substrates),^37^ and, more recently, in self-hybridized Fabry–Pérot microcavities in single crystals.^38^ These systems typically rely on thick crystals or engineered cavity architectures. In 2DLP thin films, these hybrid states can promote more efficient exciton generation and enhance the spectral selectivity of the PDs.^39^ Further, the cavity-enhanced absorption is highly advantageous for PD applications, where boosted exciton generation directly improves the PD’s photocurrent and responsivity. Planar architectures offer high sensitivity and low noise, while introducing optical cavities improves light absorption and exciton generation.^40^ Hybrid systems of 2DLP with graphene or transition metal dichalcogenides (TMDs) have been used to enhance charge transfer, enabling low-power, high-gain current detection.^41^ While most reported 2DLP PDs employ planar device architectures, such configurations typically require an external bias to drive charge extraction. In contrast, vertical PD architectures can operate in a self-powered mode, as they inherently benefit from a built-in electric field across the vertical junction, efficiently separating photogenerated carriers without any applied voltage.^42,43^ This architecture not only enables zero-bias operation but also offers sensitivity for weak light detection and energy-efficient optoelectronics. As a note, self-powered ultraviolet or broadband photodetection using materials solutions other than perovskites is a very active field of research.^44–47^

Despite these advancements, challenges remain due to the high exciton binding energy in these materials, which hinders exciton dissociation and charge transport. Additionally, organic spacer layers act as insulating barriers limiting vertical charge transport. To address these challenges, various conjugated and heteroatomic spacers have been employed to enhance the interlayer coupling and reduce dielectric mismatch.^24,48–50^ Organic spacer groups with π-conjugation have been shown to enhance charge mobility, reduce trap states, promote interlayer interactions through π–π stacking and cation-π interactions.^51^ Among the heteroatomic systems, indole and thiophene-based organic spacers have attracted attention due to their electronic delocalization and presence of electron-rich heteroatoms such as nitrogen and sulfur.^48,52^ Nitrogen-based aromatic spacers offer enhanced polarizability, charge delocalization, and tunable electronic coupling with the inorganic framework, thereby enhancing light-matter interactions.^53–55^ Additionally, indole-containing organic cations have been shown to exhibit strong cation-π and π–π interactions, which enhance charge-transport properties, facilitate prompt responses, and improve performance in photodetection applications.^51^

In this work, we report the synthesis of 2DLPs incorporating tryptammonium (TPA^+^) as the organic spacer cation. Tryptammonium offers several structural advantages, including an extended π-conjugated system, and CH-π and NH-π interaction capabilities, which may potentially enhance charge transport compared to previous layered systems. In particular, the nitrogen-containing indole ring and the terminal ammonium group in TPA-Br provide additional advantages, as the ammonium group can form strong hydrogen bonds with the inorganic [PbX_4_]^2+^ framework. Using a slow-evaporation diffusion method, TPA_2_PbBr_4_ microflakes are synthesized, and thin films are fabricated by spin coating for structural and optical characterization, as well as for device fabrication. These thin films exhibit high crystallinity and a sharp excitonic absorption peak around ∼400 nm. Due to their uniformity and refractive index close to ∼2, the 2DLP TPA_2_PbBr_4_ films also exhibit self-formed Fabry–Pérot resonances in their reflectance spectra, confirming the presence of microcavity modes that enhance light confinement and absorption, directly in spin-coated TPA_2_PbBr_4_ thin films without any additional mirrors. Strong coupling between the cavity modes and the excitons leads to a Rabi splitting of around ∼200 meV. Atomistic simulations reveal that the electronic structure of this compound features the antibonding band edges typical of lead halide perovskites, with the electronic states of TPA lying slightly away from the band gap. PDs fabricated using these TPA_2_PbBr_4_ films exhibit a photoresponse with a sharp signal onset at ∼400 nm and pronounced photoresponsivity in the UV range with low dark current (∼10^–12^ A range). Beyond planar PDs, we demonstrate photodetection from a vertical architecture that benefits from the intrinsic built-in electric field across the nanoscale thickness of the TPA_2_PbBr_4_ films, which enables them to operate as photodiodes. The robust performance, high homogeneity, and stability of our PDs under ambient conditions highlight the potential of TPA_2_PbBr_4_ for reliable and energy-efficient UV optoelectronic applications.

## 2 Results and Discussion

The tryptammonium hydrobromide (TPA-Br) salt is synthesized via a single-step synthesis following a previously reported method.^51^ Tryptamine treated with an aqueous hydrobromic acid (HBr) solution yields tryptammonium bromide (TPA-Br) as an off-white solid. The structure of the TPA-Br salt is verified using nuclear magnetic resonance (NMR) techniques (Figures S1 and S2) and further confirmed by single-crystal and powder X-ray diffraction (XRD) analyses (Figures S3, S4 and Table S1, S2), in excellent agreement with literature data.^56^ Detailed synthesis procedures are thoroughly discussed in the Experimental Section.

The TPA_2_PbBr_4_ microcrystals are synthesized using the antisolvent vapor diffusion method, following a previously reported protocol.^57^ A vial containing a 2:1 stoichiometric ratio of TPA-Br salt and PbBr_2_ in dimethylformamide (DMF) is placed in a larger vial containing dichloromethane (DCM) as an antisolvent, as shown in Scheme 1. Slow diffusion of the antisolvent into the precursor solution facilitates the slow crystallization of TPA_2_PbBr_4_. After approximately 5 days, colorless micrometre-sized crystals grew on the side of the vial. The TPA_2_PbBr_4_ perovskite crystal structure is investigated using single-crystal X-ray diffraction analysis (Figure 1a,b, Tables 1 and S3).

**Scheme 1:**
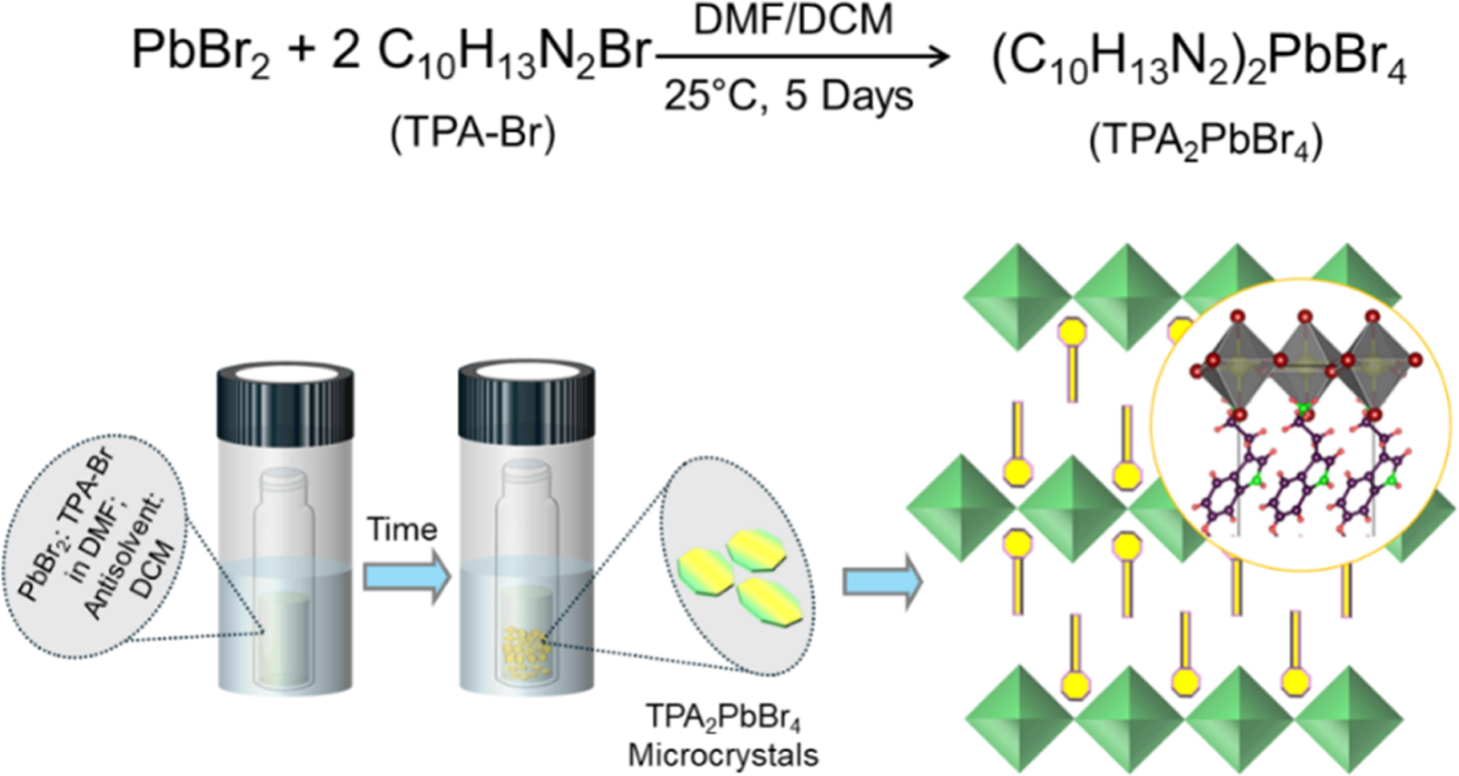
Schematic Representation of the Slow Crystallization Diffusion Method to Synthesize TPA_2_PbBr_4_

**Figure 1:**
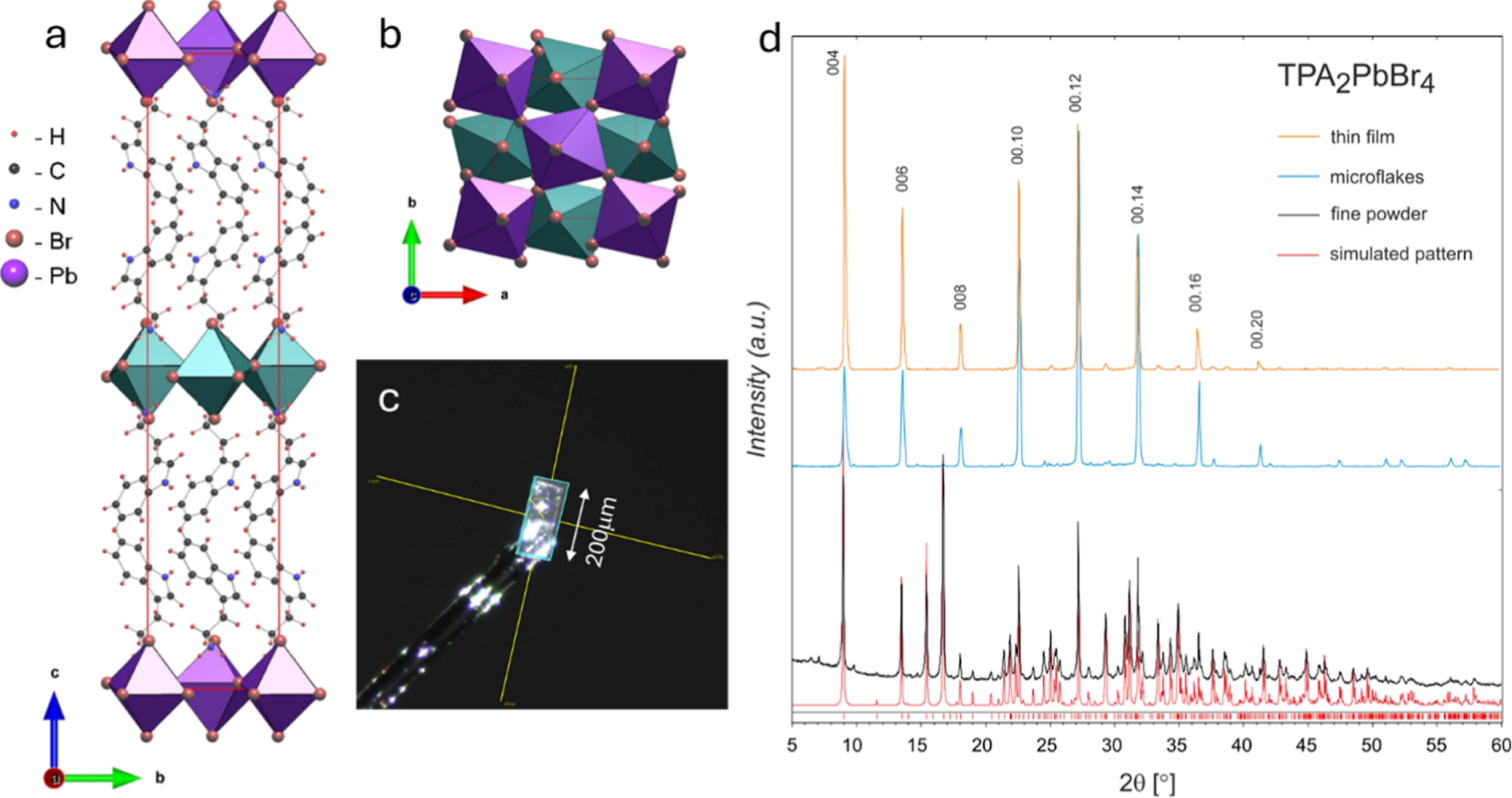
(a,b) Single crystal structure of the TPA_2_PbBr_4_ perovskite along the *c*-axis and *a*-axis. (c) Optical image of a TPA_2_PbBr_4_ microflake. (d) *p*-XRD pattern of the TPA_2_PbBr_4_ of thin film, microflakes, and fine powder, matching the corresponding simulated pattern.

**Table 1:** Selected Crystallographic Data and Structure Refinement Parameters for the TPA_2_PbBr_4_ Single Crystal

compound	TPA_2_PbBr_4_
CCDC number	2360467
empirical formula	C_20_H_26_Br_4_N_4_Pb
formula weight[g/mol]	849.28
crystal system	orthorhombic
space group (number), *Z*	*Pbca* (61), 4
*a*[Å]	8.2817(4)
*b*[Å]	8.1151(4)
*c*[Å]	39.326(2)
calculated density, ρ_calc_[g/cm^3^]	2.134
index ranges	–11 ≤ *h* ≤ 11
	–11 ≤ *k* ≤ 11 – 56 ≤ *l* ≤ 56
independent reflections	4032
	*R*_int_ = 0.1038
	*R*_sigma_ = 0.0712
data/restraints/parameters	4032/0/134
goodness-of-fit on *F* ^2^	0.993
final *R* indexes [*I* ≥ 2σ(*I*)]	*R*_1_ = 0.0429
	w*R*_2_ = 0.0680
final *R* indexes [all data]	*R*_1_ = 0.1227
	w*R*_2_ = 0.0870
largest peak/hole [eÅ^–3^]	0.94/–0.70

The compound crystallizes in the orthorhombic *Pbca* space group. The crystals adopt a 2DLP architecture, composed of corner-sharing (PbBr_6_)^4–^ octahedra and layers of TPA^+^ cations occupying the space between the two-dimensional inorganic sheets (Figure 1a,b; see the Supporting Information for more details). The inorganic layer exhibits a staggered arrangement across adjacent sheets with an interlayer distance of 19.1 Å. The optical image of a TPA_2_PbBr_4_ microflake (Figure 1c) reveals a well-defined plate-like morphology, typically observed in 2DLPs. The structure of the TPA_2_PbBr_4_ is further confirmed using powder X-ray diffraction (*p*-XRD) analysis (Figures 1d and S5). The *p*-XRD of TPA_2_PbBr_4_ thin film and microflakes exhibits sharp and well-defined diffraction peaks, with a preferred orientation along the [0 0 1] crystallographic direction, which is a characteristic feature of 2DLPs. To minimize the effect of the strong preferential orientation, the microflakes are ground prior to measurement, yielding a pattern with many additional reflections that perfectly match the simulated one. The diffraction patterns of samples in different forms (microflakes, films and fine powders) are consistent, confirming their structural identity. Notably, the *p*-XRD patterns remained unchanged after six months of storage in air at ambient conditions (Figure S6). As already mentioned before, the structure of this compound was already reported some years ago,^56^ but neither its synthesis had been disclosed nor its properties had been studied.

Temperature-dependent X-ray diffraction analysis demonstrates the structural stability of the material across the wide temperature range from 183 to 433 K (see Figure S7a,b). A gradual and slight shift of the (00*l*) diffraction peaks toward lower 2θ angles is observed with increasing temperature (Figure S7b). This shift corresponds to an increase in the (00*l*) interplanar spacing, which implies a minor expansion of the *c* lattice parameter upon heating. This behavior is consistent with typical thermal expansion in layered perovskites, where the out-of-plane direction can expand due to weak van der Waals interactions.^58^ The in situ temperature-dependent XRD analysis confirms that the 2D perovskite exhibits good thermal stability, with only minimal reversible thermal expansion changes.

The morphology and surface topology of TPA_2_PbBr_4_ in various forms are investigated using scanning electron microscopy (SEM), atomic force microscopy (AFM), and optical profilometry. Figure 2a shows an SEM image of the TPA_2_PbBr_4_ microflakes, with sizes varying up to 200 μm and exhibiting a layered structure. A representative SEM image of a TPA_2_PbBr_4_ thin film (Figure 2b), obtained by spin-coating a mixture of precursors on a quartz substrate, reveals a dense network of randomly oriented platelets, indicating a polycrystalline morphology. SEM-EDS elemental mapping of TPA_2_PbBr_4_ in both microflake and thin-film forms confirms a uniform distribution of Pb and Br across the samples. The atomic ratio of Pb/Br is 1:3.7 and 1:3.9 for the microflakes and thin film, respectively, in accordance with the expected Pb/Br stoichiometric ratio of the 2DLPs (1:4). This further denotes the formation of the layered TPA_2_PbBr_4_ structure in both microflake and thin-film forms (Figures S8, S9 and Tables S4, S5, also a cross-section of the TPA_2_PbBr_4_ thin film of Figure 2b is presented in Figure S10). The AFM image in Figure 2c demonstrates uniform surface coverage, with a root-mean-square (RMS) roughness of 2.5 nm. While the large crystals require approximately 5 days to grow, the thin film dries on a time scale of minutes, making the formation of much smaller crystallites highly plausible. The AFM morphology is consistent with the top-view surface SEM image of the TPA_2_PbBr_4_ thin film. The AFM surface and the height profile of the TPA_2_PbBr_4_ thin film are depicted in Figure S11. Films with optimal homogeneity and surface roughness are subsequently used for the optical and electrical experiments discussed later. The optical image of a uniform TPA_2_PbBr_4_ thin film is presented in Figure S12.

**Figure 2:**
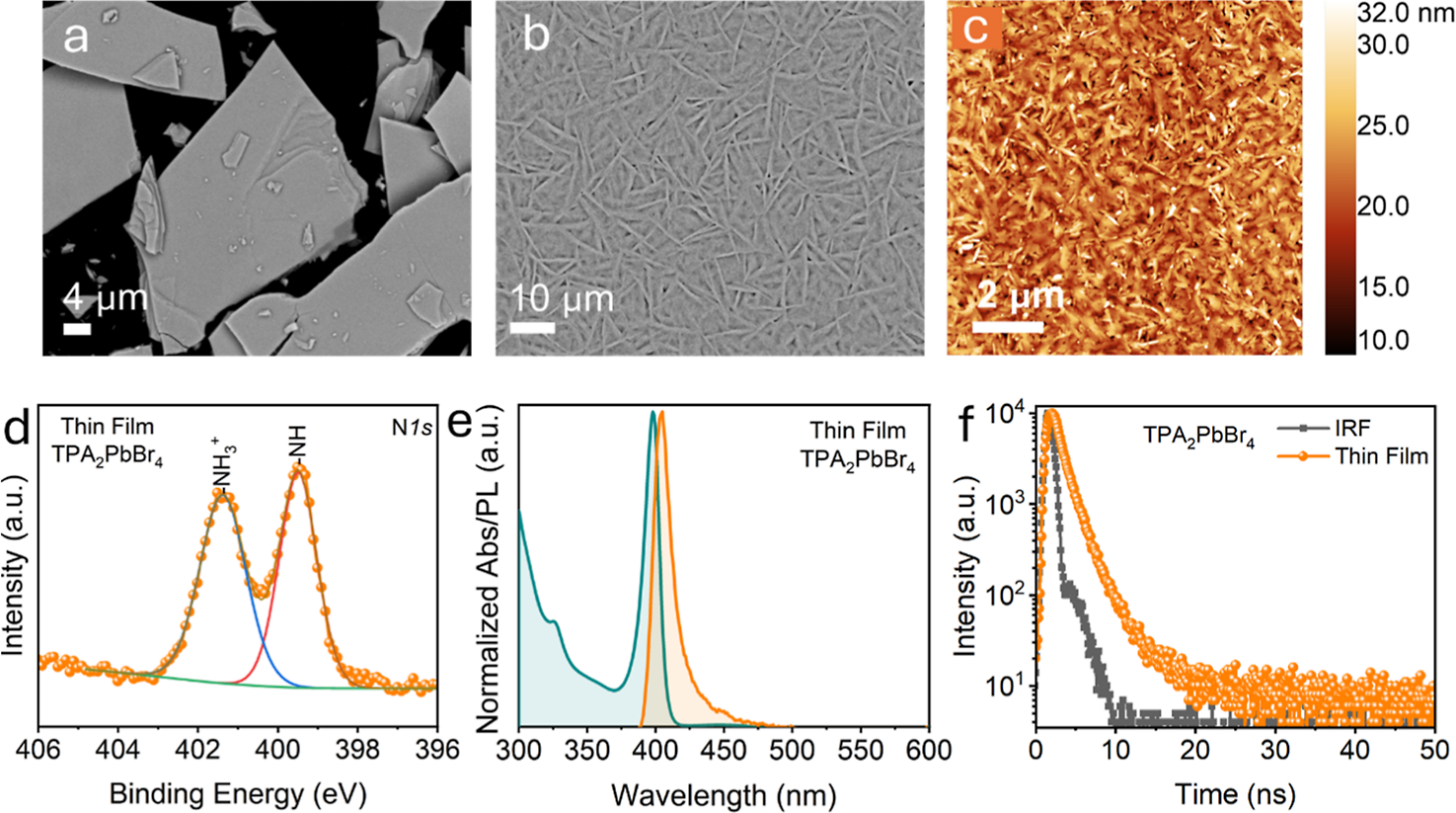
SEM image of the TPA_2_PbBr_4_ perovskite, (a) microcrystal powders and (b) thin film. (c) AFM image of the TPA_2_PbBr_4_ thin film. (d) N 1s XPS spectrum of the TPA_2_PbBr_4_ thin film in the N 1s region. (e) UV–visible absorption (blue) and photoluminescence (orange) spectra of the TPA_2_PbBr_4_ thin film. (f) PL decay of the TPA_2_PbBr_4_ thin film.

We also perform X-ray photoelectron spectroscopy (XPS) measurement. The N 1s XPS spectrum of TPA_2_PbBr_4_ thin film reveals two distinct peaks at 401.4 and 399.5 eV, corresponding to the protonated amine group (–NH_3_^+^) and the indole-based –NH group, respectively (Figure 2d). The higher binding energy peak indicates a positively charged nitrogen TPA^+^ spacer, whereas the lower energy peak denotes partial coordination due to an NH-π interaction, as observed in the crystal structure. The compositional analysis of TPA_2_PbBr_4_ obtained from XPS is summarized in Table S6.

The optical properties of TPA_2_PbBr_4_ perovskite are investigated in thin film and microflakes using UV–visible absorption and photoluminescence (PL) spectroscopy. The absorption spectra of the polycrystalline thin film feature strong, narrow band excitonic peaks centered at 398 nm, and the microflakes show an excitonic feature at ∼396 nm, respectively (Figures 2e and S13). The optical band gap (*E*_g_) is 2.9 eV, as derived from the Tauc plot in Figure S14. Compared to TPA_2_PbBr_4_ microflakes, which exhibit a broader PL emission peak at 420 nm, the TPA_2_PbBr_4_ thin film shows a blue-shifted PL peak at 407 nm. The broader emission in TPA_2_PbBr_4_ microflakes can be ascribed to trap states or edge emission in TPA_2_PbBr_4_, resulting from the electron–phonon coupling at the surface.^59^ Time-resolved PL decay measurements reveal a slightly longer PL lifetime for the TPA_2_PbBr_4_ microflakes, ∼3.2 ns (Figure S15), compared to that of the thin film, ∼2.18 ns (Figure 2f and Table S7).

We obtained highly homogeneous films as demonstrated by the SEM and AFM images (see Figure 2b,c). Such homogeneous films sustain Fabry–Pérot cavity modes^60^ without the need of additional mirror layers, as evidenced by the reflectance spectra reported in Figures 3a and S16. We observe a series of minima that are not equidistant in energy due to the increase of refractive index toward the semiconductor band gap.^34^ From the line width of the minima (λ/Δλ), we extract a *Q* factor of around 10 (at 650 nm) for the cavity formed by the perovskite film. Furthermore, homogeneous films enable the measurement of their polarized, angle-dependent reflectance by spectroscopic ellipsometry (Figures S17 and S18), which allows the derivation of the real and imaginary parts of the complex refractive index function, as shown in Figure 3b. The real part of *n* is 1.74 in the long wavelength limit and increases toward the singularity at the band edge to a maximum value of 2.25. The imaginary part corresponds to the losses and resembles the material’s absorbance. We observe a very good agreement between the observed sharp peak and the excitonic resonance in the optical absorption spectrum, as shown in Figure 2e. Interestingly, the quality of the self-sustained cavity of the perovskite thin films is sufficient to achieve strong light-matter coupling. Figures 3c and S19 show the hybridization of the cavity modes and exciton resonances into polariton branches (white lines), from which we derive a Rabi splitting of 200 meV (at *k* = 20 μm^–1^). We note that the line width of the polariton resonances at the band gap is significantly sharper than that of the cavity (evaluated far below the band gap) due to the excitonic contribution to the polariton. These measurements highlight the high quality of the TPA_2_PbBr_4_ thin films, making them highly appealing for optoelectronic applications that require tunable and narrowband responses.

**Figure 3:**
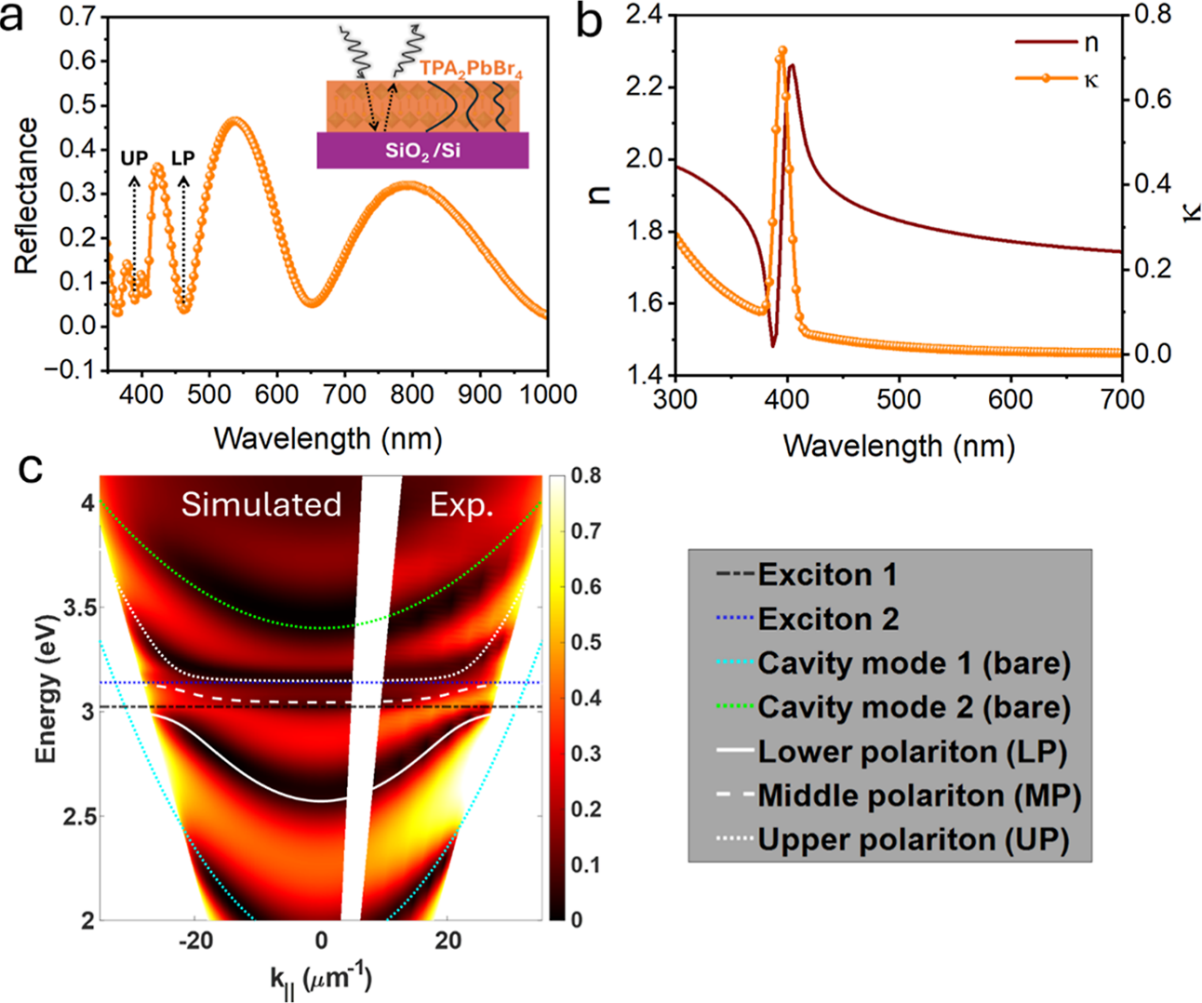
Optical properties of the TPA_2_PbBr_4_ thin films. (a) Reflectance under normal incidence with a series of minima that can be associated with the confined cavity modes (upper (UP), and lower (LP) polaritons). The inset illustrates the open cavity formed by the perovskite thin film that sustains a series of resonant modes. (b) Spectral dispersion of the real (*n*) and imaginary (κ) part of the complex refractive index obtained by spectroscopic ellipsometry. (c) Color-plot of the calculated (left) and measured (right) s-polarized reflectance intensity versus in-plane wave vector and energy of the incident light. The strong light-matter coupling leads to the formation of upper and lower polariton branches, depicted by the white lines. Details on the simulations are reported in the Experimental Section 4.4.7 and p-polarised data in Figure S19.

Density functional theory (DFT) simulations with the projector augmented wave method^63–65^ are performed to gain insights into the electronic structure of TPA_2_PbBr_4_. The band gap obtained with the HSE06 hybrid functional^66^ is 2.5 eV, in fairly good agreement with the experimental estimate, the discrepancy being in the range typically observed for (hybrid) DFT functionals.^67,68^ It has a direct character (Figure S20), thereby allowing efficient radiative recombination that leads to the observed photoluminescence (Figure 2e). The band edge states exhibit the typical features of lead halide perovskites.^69^ In particular, the valence and conduction bands are dominated by the halogen (Br) and Pb atoms, respectively, both bands being of antibonding character (Figure 4). Interestingly, above/below the conduction/valence states, electronic states relative to the molecular orbitals of the tryptamine rings are found (Figure 4). These orbitals have an energy gap of 3.5–4.5 eV, depending on the adopted level of theory (see Experimental Section). Considering the possible DFT uncertainty (e.g., neglecting the exciton binding energy), it is conceivable that the absorption peak around 320 nm (Figure 2e) arises from the excitation of organic molecules. Once the molecules are excited, the carriers can either recombine non radiatively or transfer to the inorganic layers (given the absence of additional PL peaks), where they radiatively recombine.

**Figure 4:**
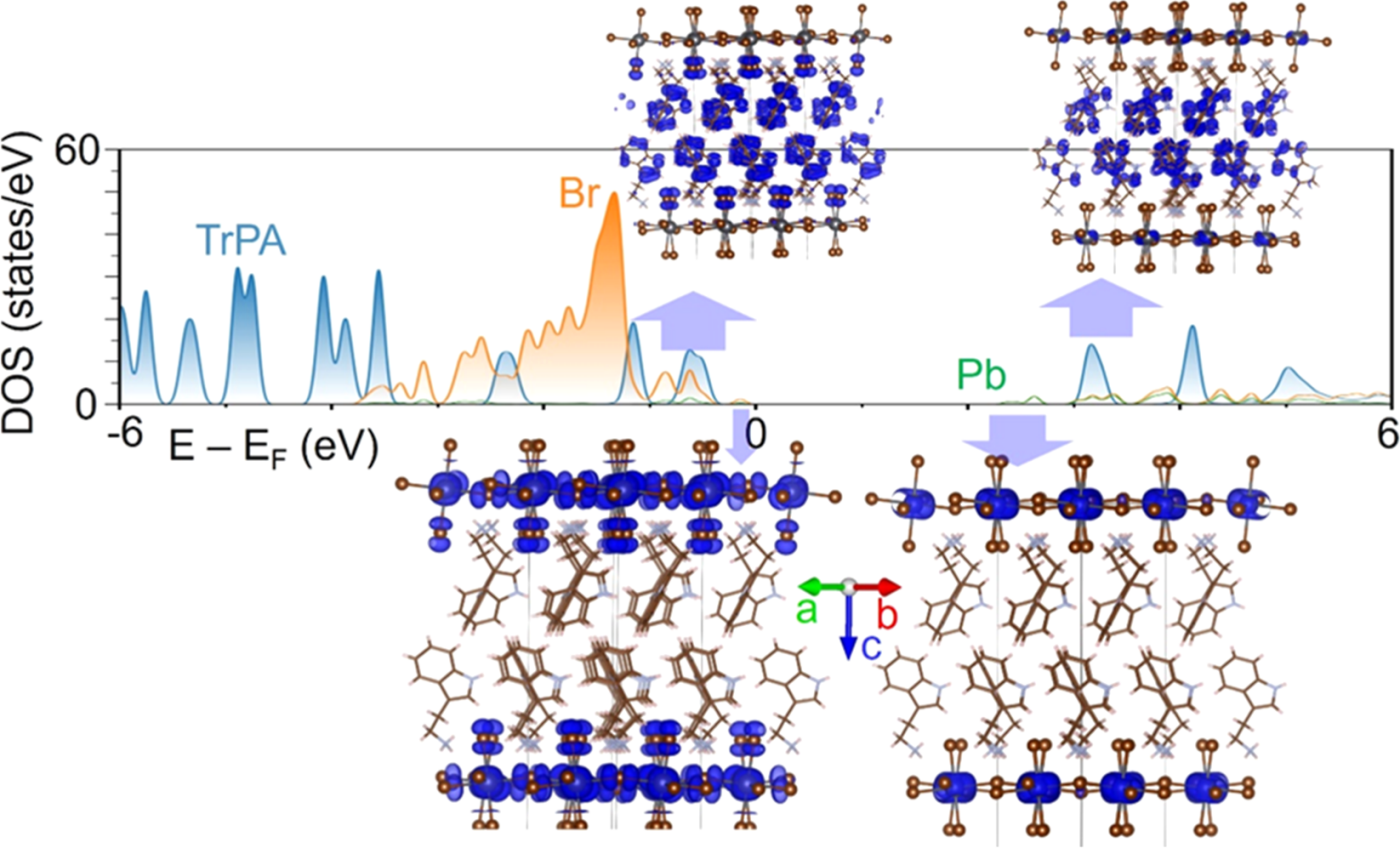
Density of states (DOS) and partial electron density of selected DOS energy windows obtained with the PBE functional^61^ neglecting spin–orbit coupling (SOC; the DOS obtained with the HSE06 functional including SOC is reported in Figure S20). The partial electron density is obtained by evaluating the electron density adopting only the crystal orbitals within a selected DOS region, thereby representing the real-space distribution of electronic states. It is represented by blue isosurfaces on half-unit cells in the images above and below the DOS. Structures and partial electron densities are drawn with VESTA software.^62^

The optoelectronic performance of the TPA_2_PbBr_4_-based PD is evaluated under various illumination powers to assess their photoresponse characteristics under ambient conditions. We fabricated a planar-type PD using Si–SiO_2_/TPA_2_PbBr_4_/Au architecture (Figure 5a). The band alignment of the TPA_2_PbBr_4_ layer, as determined by UPS analysis and optical absorption spectra, is illustrated in Figure S21 (see also Table S8). The voltage (*V*_bias_) is applied across the drain and source contact as shown in the schematic of Figure 5a. The current–voltage (*I–V*) characteristics of the device, under dark and illuminated conditions at varying power densities (all at λ = 385 nm) are shown in Figure 5b. The black curve represents the dark current, which is significantly low, in the nanoampere (nA) range, indicating a low density of shallow traps and defects that could contribute to carrier transport.^70^ Under illumination, the current increases markedly with power density, exhibiting higher values at increased light power as shown in Figure 5b. This behavior is consistent with that of conventional semiconductors and indicates efficient carrier collection, as well as the photoconductive nature of the TPA_2_PbBr_4_ device. Time-dependent dynamic photocurrent measurements with sequential increases in light power are shown in Figure 5c. In each illumination step, the current increases and stabilizes quickly across all light intensities, then drops sharply upon switching off, demonstrating a stable response over a large range of power densities. This confirms the wide dynamic range of light detection in TPA_2_PbBr_4_-based PDs (Figure 5c). A photograph of the device’s interdigitated electrode configuration is shown in Figure S22. Figure 5d highlights the stability and reproducibility of the photoresponse under continuous on/off cycling at a light power density of 6.05 mW/cm^2^. Over repeated ON/OFF switching, the photocurrent maintains a consistent amplitude and exhibits reproducible switching behavior, without any sign of degradation, underscoring the reversibility of the photoinduced charge transport. These results demonstrate that TPA_2_PbBr_4_-based PDs exhibit highly stable photoresponse under UV excitation.

**Figure 5:**
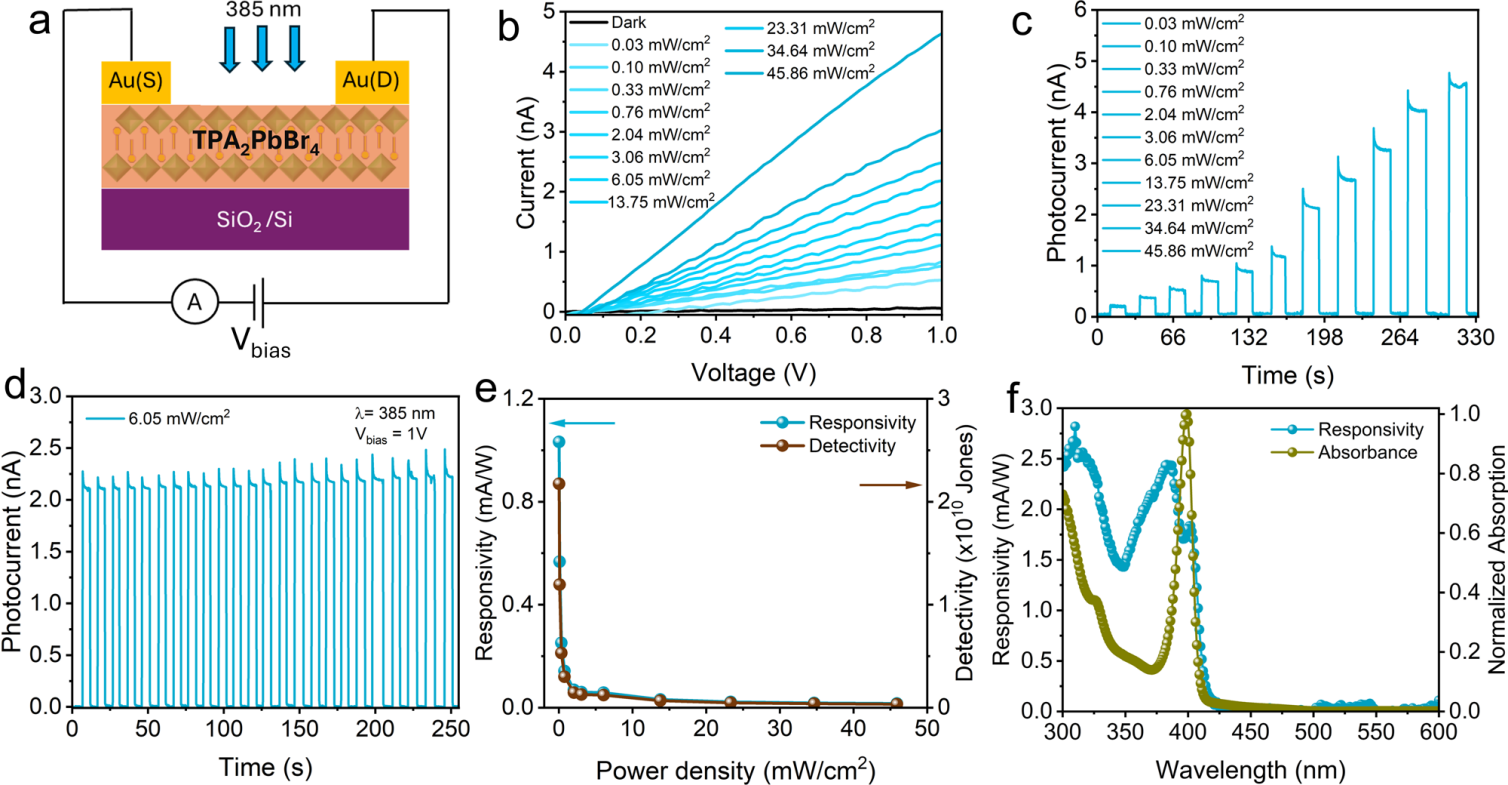
Planar TPA_2_PbBr_4_ PD device characterization. (a) Device geometry. (b) *I–V* characteristics at different light intensities. (c) Photoresponse under illumination with a 385 nm LED at different power densities. (d) Stability curve under on/off switching of 385 nm LED light illumination. (e) Responsivity and detectivity as a function of optical power density (385 nm) at a fixed bias voltage of 1 V. Active device area is 0.0064 cm^2^. (f) Wavelength-dependent responsivity at 3 V and absorption spectra.

To evaluate the performance of the 2DLP TPA_2_PbBr_4_ PDs, the key parameters of the PDs, such as responsivity (*R*), detectivity (D*), and response time, are evaluated. The responsivity (*R*) and the detectivity (D*)^71–74^ of the device are calculated using the standard formulas

1 $$R=\frac{I_{ph}}{P}$$

2 $$D^{*}=\frac{R\sqrt{A}}{\sqrt{2eI_{d}}}$$

where *I*_ph_ is the photocurrent, *P* is the incident light power, *A* is the active area of the device, *e* is the electronic charge, and *I*_d_ is the dark current. The *R* and D* values as a function of the power density are plotted in Figure 5e (see Supporting Information CS1 for complete details of calculating *R* and D*). We find a maximum responsivity of approximately 1 mA/W for the lowest illumination power at a 1 V bias. As we increase the power of the 385 nm source, the responsivity of the TPA_2_PbBr_4_ PDs decreases, indicating the generation of excess carriers that are not efficiently harvested.^75^ Figure 5f demonstrates the close overlap of the spectral responsivity of the planar PD plotted alongside the optical absorption spectrum of the TPA_2_PbBr_4_ thin film. For this spectral responsivity measurement, an Xe lamp coupled to a monochromator serves as the light source, and the device is biased at 3 V. We find close alignment of the onset in photoconductivity with the onset in absorption and additionally observe two bands of high responsivity (with around 2.5 mA/W max values) that correspond to the band edge (385 nm) and the spectral region around 320 nm. This spectral response confirms the suitability of the TPA_2_PbBr_4_ film for UV photodetection. The power-dependent photocurrent manifests a logarithmic behavior versus power density (Figure S23). The exponent value less than unity suggests the presence of trap states that influence carrier transport dynamics, likely decreasing the photocurrent gain.^76^ This behavior is characteristic of semiconductor-based PDs, arising from the intrinsic processes of exciton pair generation, trapping, and recombination. The curve follows a linear trend with the power exponent of *a* = 0.43, as fitted by the equation

3 $$I_{ph}\propto P^{a}$$

The response time is determined using the 90%–10% thresholds, as depicted in Figure S24 (derived from Figure 5c at a power density of 3.06 mW/cm^2^). The measured rise time is approximately 0.17 s, while the fall time is 0.25 s. The comparatively longer fall time is attributed to the trap states.

Figure S25 shows that the TPA_2_PbBr_4_ planar PD maintains stable, reproducible photoresponse for more than months, with clear ON/OFF switching under 385 nm illumination across a wide range of power intensities, confirming long-term operational stability at ambient conditions.

In addition to the planar configuration, we fabricated a vertical PD architecture, FTO/TPA_2_PbBr_4_/Au (Figures 6a and S26), in which the 2DLP film is sandwiched between FTO, serving as the transparent bottom electrode, and Au as the top contact, with a large device active area of 0.16 cm^2^. A schematic energy band alignment diagram for the vertical photodetector is illustrated in Figure S27. Vertical geometry facilitates efficient carrier collection (thanks to the submicron-thick active layer) and enables self-powered operation due to the built-in electric field generated by the asymmetric FTO-TPA_2_PbBr_4_ and TPA_2_PbBr_4_–Au junctions, eliminating the need for an external bias. The time-dependent photocurrent response at 0 V under varying illumination intensities (Figure 6b) shows a stepwise increase in the photocurrent with increasing power density, confirming the device’s stable and reproducible photoconductive behavior even under self-bias operation. The dark current of the TPA_2_PbBr_4_ vertical PD at self-bias operation is depicted in Figure S28. The responsivity and the detectivity of the vertical devices reach values of 0.65 mA/W and 2.48 × 10^10^ Jones, respectively, at low incident light power^77^ (Figure 6c) (see Supporting Information CS2 for details of calculation of the TPA_2_PbBr_4_ thin film acts as a self-formed Fabry–Pérot cavity, increasing the exciton generation efficiency, and (ii) the strong built-in potential or internal electric field at the asymmetric FTO/TPA_2_PbBr_4_/Au contact junction promotes efficient charge separation without any external bias and minimizes recombination loses. Although *R* and D* values obtained for our PDs are modest compared to the highest-reporting 2DLP PDs reported in the literature (Table S9),^28,78–80^ it is important to emphasize that TPA_2_PbBr_4_-based vertical PDs operate without addition of charge-selective transport layers. Achieving stable and wavelength-selective photodetection at zero bias with a large active area (0.16 cm^2^) demonstrates the intrinsic capability of TPA_2_PbBr_4_ perovskite for efficient charge-carrier separation and transport. Figure S29 illustrates the stable photocurrent response with negligible fluctuations during continuous operation. Reproducible photoresponse under continuous cycling shows no signs of degradation, confirming the reversibility of photoinduced charge-transport under self-powered operation. To further evaluate the operational stability, the photocurrent of the vertical PD was continuously monitored under 385 nm illumination at 0 V bias (Figure S30). The photocurrent decreases during the first ∼20 min of continuous UV exposure and subsequently stabilizes, remaining nearly constant throughout the measurement, indicating good operational stability under prolonged UV irradiation.

**Figure 6:**
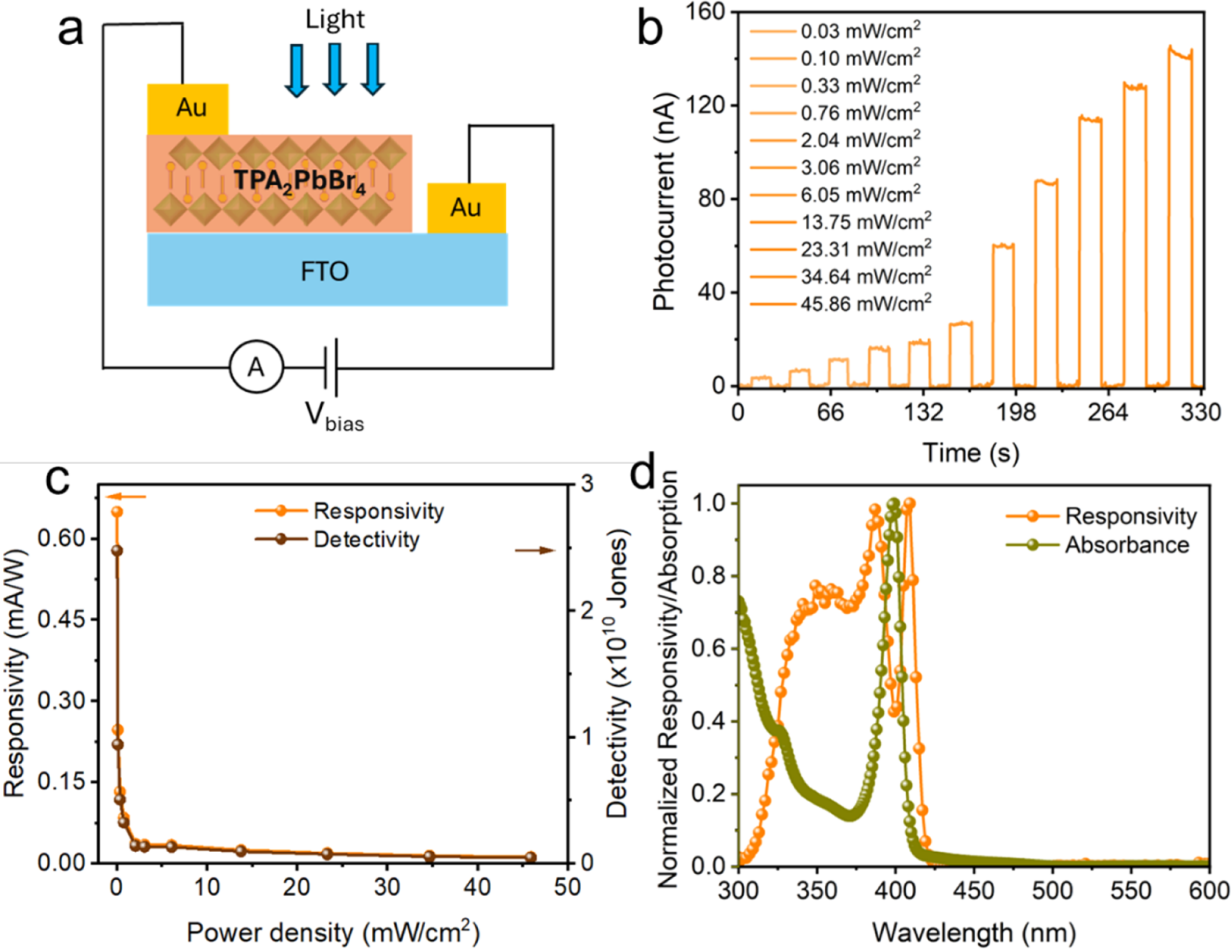
Vertical TPA_2_PbBr_4_ PD device characterization. (a) Architecture of the TPA_2_PbBr_4_ vertical PD device. (b) Photoresponse under 385 nm LED with different power intensities. (c) Responsivity and detectivity versus power density in self-biased mode (bias = 0 V) and excitation wavelength of 385 nm. (d) Normalized spectral responsivity at 0 V and absorption spectra.

The wavelength-dependent responsivity (Figure 6d) reveals a double peak at the absorption edge of TPA_2_PbBr_4_ perovskite. Interestingly, the lower peak coincides in energy with the “Exciton1 position” peak in our modeling of the strong light matter interaction in Figure 3c. This lower energy exciton band could stem from long-lived edge states as reported by Li et al.,^81^ which result from out-of-plane strain at the borders of the 2D perovskite microcrystals. In the vertical PD configuration, the charge carrier collection pathways are significantly shorter compared to the horizontal one, therefore these edge states with long lifetimes should have a strong impact in photocurrent. In combination with microcavity effects of the thin film^82^ (see polaritonic features in Figure 3a–c that enhance the contribution of edge states^71^), the attribution of the low energy peak to such edge states is therefore plausible. The vertical TPA_2_PbBr_4_ perovskite PD devices showed good stability, confirmed by EQE characterization measured after approximately two months of storage in a drybox (Figure S31) that showed negligible changes.

## 3 Conclusion

We have successfully synthesized and comprehensively characterized TPA_2_PbBr_4_ two-dimensional perovskites, employing tryptammonium as a π-conjugated organic spacer cation. The material exhibits excellent crystallinity, a well-defined layered structure and thermal phase stability, as confirmed by single-crystal and temperature-dependent XRD analyses. Its strong excitonic absorption and narrow PL emission highlight the intrinsic optical quality of the TPA_2_PbBr_4_ structure. Compared with previous studies that rely on thick single crystals or mirror-assisted cavities for strong coupling, we observe self-formed Fabry–Pérot microcavities directly in our TPA_2_PbBr_4_ thin films, thereby enhancing light confinement within the active layer. Planar PDs fabricated from TPA_2_PbBr_4_ films exhibit UV selectivity with a sharp onset at approximately ∼400 nm, a low dark current, and a stable photoresponse under ambient conditions. Furthermore, the vertical PDs fabricated with TPA_2_PbBr_4_ films leverage the built-in potential across the vertical junction, enabling self-powered operation with enhanced responsivity and detectivity under UV illumination at zero external bias. The vertical PDs, even with a large device area of 0.16 cm^2^ for TPA_2_PbBr_4_ films, exhibit a responsivity of 0.65 mA/W in the UV and a D* value of 2.48 × 10^10^ Jones under zero-bias mode, without incorporating any charge-selective or transport layers. The ability to operate without external bias, combined with the material’s intrinsic stability and sharp excitonic absorption, demonstrated how microcavity-enhanced light confinement enhances carrier generation, highlighting TPA_2_PbBr_4_ as a promising material for UV photodetectors.

## 4 Experimental Section

### 4.1 Materials

Lead bromide (PbBr_2_ 99.99%), tryptamine (TPA, 90%), dimethylformamide (DMF, 97%), dichloromethane (DCM, anhydrous, 90%), diethyl ether (HPLC grade, 99.9%), hydrobromic acid (HBr), dimethyl sufoxide-d6 (DMSO-*d*_6_, 99.9 atom % D), are purchased from Sigma-Aldrich. Gold pellets were purchased from Ted Pella for use in evaporation. Silicon oxide substrates (300 nm oxide, diced) and conducting glass substrates were purchased from Ossila. Unless otherwise stated, all the materials are used without further purification.

### 4.2 Syntheses

#### 4.2.1 Synthesis of Tryptammonium Hydrobromide: C_10_N_2_H_12_.HBr; (TPA-Br)

Tryptamine (2 g, 12.5 mmol) is dissolved in 10 mL of ethanol and stirred under an ice bath. Aqueous hydrobromic acid (HBr, 1.01 mL, 12.5 mmol) is added dropwise to the solution, which is then stirred for 4 h. After the reaction is complete, the mixture is concentrated under reduced pressure. The compound is further washed with diethyl ether (5 times, 20 mL each) using a Buchner funnel, then dried under vacuum at room temperature to obtain the final product as the TPA-Br salt. A white solid product is obtained, weighing 1.81 g, with a yield of 60%. ^1^H NMR and ^13^C NMR spectroscopy are used to ascertain the chemical structure and purity of the TPA-Br salt. ^1^H NMR (600 MHz, DMSO-*d*_6_) δ: 10.97 (s, 1H), δ: 7.83 (s, 3H), δ: 7.56 (d, 1H), δ: 7.38 (d, 1H), δ: 7.24 (d, 1H), δ: 7.10 (t, 1H), δ: 7.09 (t, 1H), δ: 2.99–3.09 (m, 4H). ^13^C NMR (150 MHz, DMSO-*d*_6_) δ: 136.30, 126.80, 123.41, 121.18, 118.48, 118.09, 111.55, 109.35, 39.94, 23.10.

#### 4.2.2 Growth of Two-Dimensional Layered Hybrid Perovskite, TPA_2_PbBr_4_; (C_10_H_13_N_2_)_2_PbBr_4_ Crystals

A clear solution is prepared by dissolving stoichiometric amounts of various TPA-Br salts (0.6 mmol) and lead bromide (PbBr_2_, 0.3 mmol) in a 5 mL small vial, to which anhydrous dimethylformamide (DMF, 1 mL) is added and kept at 80 °C under constant stirring for 2 h. After that, the solution is allowed to cool, and a small vial containing the precursor solution is inserted into a larger vial (20 mL) containing 5 mL of dichloromethane (DCM) as the antisolvent. Over time of 5 days, the DCM evaporates and diffuses into the DMF solution of the salt mixture. This causes the liquid in the small vial to rise, and slowly, the crystals begin to deposit, allowing us to obtain the small microcrystals.

### 4.3 Device Fabrication

#### 4.3.1 Photodetector Fabrication Using TPA_2_PbBr_4_ Layered Perovskites

First, the SiO_2_ (300 nm oxide, diced) and FTO substrates are sequentially ultrasonically cleaned using a Hellmanex (2%) cleaning solution, DI water, acetone, and isopropanol, followed by drying with a nitrogen flow. Subsequently, the substrates underwent O_2_-plasma treatment for 3 min to achieve a clean surface. For the photodetector (PD) devices, a TPA_2_PbBr_4_ solution in DMF is spin-coated onto the treated SiO_2_ substrate at 3000 rpm for 30 s and then annealed at 80 °C for 15 min under ambient conditions. Then, a metal electrode (Au) is deposited in a vacuum chamber through an interdigitated shadow mask at a base pressure of 5 × 10^–6^ Torr. The thickness of the deposited layers is monitored to be approximately 100 nm.

The vertical PDs are fabricated on FTO substrates by spin-coating a TPA_2_PbBr_4_ solution in DMF at 3000 rpm for 30 s, followed by annealing at 80 °C for 10 min under ambient conditions. Then, a metal electrode (Au) is deposited in a vacuum chamber through a shadow mask with an area of 0.16 cm^2^ at a base pressure of 5 × 10^−6^ Torr. The thickness of the deposited layers is monitored to be approximately 100 nm.

### 4.4 Methods

#### 4.4.1 Photodetector Characterization

The device’s electrical characterization is conducted using a Suss MicroTec and Janis probe station with an Olympus microscope. DC voltage and current measurements are performed using a Keithley 2614B source meter. Thor Lab LED from 385 nm is used to excite the sample. A UV card from Thor Laboratories is used to visualize the LED spot for 385 nm light. Power measurements are carried out using a Thor Lab power meter (PM130D). All measurements are performed under ambient temperature conditions.

The responsivity measurement of the vertical PDs is acquired with a Newport Merlin radiometry system equipped with a xenon lamp, an optical beam chopper, a Newport Cornerstone 260 monochromator, an Oriel Merlin digital lock-in amplifier/chopper driver and a transimpedance amplifier (Newport 70710). The photocurrent signal of the PDs is collected under a chopped monochromated beam excitation with no external voltage bias applied. The photocurrent is fed into the transimpedance amplifier for current-to-voltage conversion and then sent to the lock-in amplifier for detection. TracQ software is used to control the system and to acquire the reference/device spectra. The reference spectrum is collected via a calibrated silicon diode with an integrated transimpedance amplifier (Newport 70356).

#### 4.4.2 X-ray Diffraction Analysis

X-ray diffraction (XRD) measurements are conducted in Bragg–Brentano geometry using a third-generation Empyrean diffractometer (Malvern-PANalytical, Malvern, UK). The system has a 1.8 kW CuKα ceramic X-ray source operating at 45 kV and 40 mA. It features automated PreFIX iCore-dCore optical modules for incident and diffracted beam paths. Data acquisition is performed using a PIXcel3D area detector.

Temperature-dependent XRD measurements are conducted using an Anton Paar TTK 600 chamber, which enables measurements in nonambient conditions ranging from −190 to 600 °C. The heating and cooling rates are set to 2 °C/min, and each XRD scan is preceded by a 20 min temperature stabilization period to ensure thermal equilibrium. Samples are analyzed under an inert nitrogen atmosphere (flow rate: 5 Nl/h) to prevent oxidation or other atmospheric interactions.

The TPA_2_PbBr_4_ samples for diffraction measurements are prepared by concentrating the solution under nitrogen and drop-casting the dispersions on a zero-diffraction Si substrate.

#### 4.4.3 Optical Absorption and Photoluminescence Spectroscopy

Optical absorption and photoluminescence spectra are recorded using a Varian Cary 5000 UV–vis spectrophotometer and an Edinburgh FLS900 spectrophotometer, with an excitation wavelength of 375 nm for all samples. The samples are prepared by spin-coating the TPA_2_PbBr_4_ perovskite solution on quartz substrates (20 mm × 15 mm) and placing the microflakes between two quartz substrates.

#### 4.4.4 Time-Correlated Single-Photon Counting

Time-resolved photoluminescence spectra (TRPL) at room temperature are obtained using an FLS900 Edinburgh spectrophotometer, and the PL decay traces are measured with a pulsed laser diode (λ_ex_ = 375 nm, 10 MHz repetition rate, 60 ps pulse width) and fitted with a two-exponential decay function.

#### 4.4.5 X-ray Photoluminescence Spectroscopy

X-ray photoelectron spectroscopy (XPS) measurements are carried out through a Kratos Axis Ultra DLD spectrometer (Kratos Analytical Ltd.) with a monochromated Al K_α_ X-ray source (*h*ν = 1486.6 eV) operating at 20 mA and 15 kV. Specimens are prepared by dropping a concentrated NCs solution into toluene onto a highly ordered pyrolytic graphite (HOPG, ZYA grade) substrate. The wide scans are collected over an analysis area of 300 × 700 μm^2^ at a photoelectron pass energy of 160 eV and an energy step of 1 eV. In contrast, high-resolution spectra of Cs 3d, Pb 4f, Br 3d, and C 1s are collected at a photoelectron pass energy of 20 eV and an energy step of 0.1 eV. A takeoff angle of 0° with respect to the sample’s normal direction is used for all analyses. The slight differential electrical charging effects (less than 0.5 eV) are observed on all samples that are not neutralized. The irradiation of an APbX_3_-like perovskite with an electron beam is known to induce the desorption of halogen species and the nucleation of metallic Pb particles.^85^ The spectra have been referenced to the adventitious carbon 1s peak at 284.8 eV. The spectra are analyzed with the Casa XPS software (Casa Software Ltd., version 2.3.24),^86^ and a residual background is eliminated by the Shirley method across the binding energy range of the peaks of interest. The relative atomic concentrations are then estimated using the specific function in the Casa XPS software. The slight differential electrical charging effects (less than 0.5 eV), observed on all samples, are not neutralized.

#### 4.4.6 Ultraviolet Photoelectron Spectroscopy

The ultraviolet photoelectron spectroscopy (UPS) measurements are performed using a He I (21.22 eV) discharge lamp, fitted in the same chamber used for XPS analyses, on an area of 55 μm in diameter, at a pass energy of 10 eV and with a dwell time of 100 ms. The energy levels within the equipment and UPS-based calculations are described as follows. The work function, φ, i.e. the position of the Fermi level versus the vacuum level, is determined for each sample from the position of the secondary electron cutoff in the UPS spectrum, using the following equation: φ = *hv* – *E*_0_, where *h*ν is the source energy (21.22 eV for He I photons) and *E*_0_ is the secondary electron cut off. The position of the valence band maximum (VBM) versus the vacuum level, i.e., the ionization energy, E_ion_, is determined from the width of the entire UPS spectrum, according to the following equation: *E*_ion_ = *hv* – (*E*_0_ – *E*_1_) = *hv* – *E*_0_ + *E*_1_, where *E*_1_ is the position of the VBM concerning the zero (Fermi) level.^77^ The values *E*_0_ and *E*_1_ are determined in the UPS spectrum through the background functions “Edge Up” and “Edge Down” in the Casa XPS software. The error bar associated with this procedure is estimated to be equal to 0.2 eV.

#### 4.4.7 Reflectance Measurements for 2D Layered TPA_2_PbBr_4_ Perovskite

Optical characterisations: reflectance measurements on thin films are conducted using a custom-built optical setup featuring integrated in situ imaging. To ensure distortion-free reflectance data, a collimated beam from a broadband light source (AvaLight-DH-S, Avantes) is directed onto the sample through a 20× reflective objective (Thorlabs) with a numerical aperture of 0.5. This same objective also collected the reflected light, which is then transmitted to a fiber-coupled spectrometer (AvaSpec2048, Avantes). For normalization, reflectance spectra are referenced against a signal obtained from a high-reflectivity silver mirror (Thorlabs). A camera (Thorlabs) is integrated into the optical path enabled real-time visualization of the sample surface, facilitating accurate alignment and assessment of film quality. To determine the refractive index, spectroscopic ellipsometry is performed using a Woollam M2000 Ellipsometer across the 300–600 nm wavelength range. Angle-resolved reflectance measurements for both S-and P-polarized light are acquired at incidence angles ranging from 20° to 80°, in 10° increments.

The scattering matrix modeling (SMM) is employed to compute the (angle-dependent) the reflectance spectra using the measured complex refractive index of the perovskite film and its layer thickness measured by AFM. The angle is then converted to an in-plane *k*-vector, and the cavity dispersion is calculated as

$$E_{ph}\left(k\right)=E_{c0}+{\alpha k}^{2}$$

where *E*_c0_ is the mode energy at normal incidence, and α describes the curvature obtained in the SMM simulations.

To calculate the anticrossing and Rabi splitting, we use a coupled Oscillator Model with Two Excitons (3 × 3 Hamiltonian), which correctly produces three hybridized polariton branches for modes that couple to excitons.

$$H=\left(\begin{array}{lll} E_{c0} & g1 & g2\\ g1 & Eexc1 & 0\\ g2 & 0 & Eexc2 \end{array}\right)$$

The simulation parameters are:

(a) Film thickness 210 nm (obtained from AFM)
(b) *E*_exc1_ = 3.024 eV and *E*_exc1_ = 3.14 eV (obtained from optical spectra), and their oscillator strengths are implemented as fitting parameters in the coupling terms: *g*1 = 100 meV and *g*2 = 60 meV (Tables 2 and 3).

**Table 2:** S-Pol

cavity mode	*E*_c0_ (eV)	α (eV·μm^2^) α × 10^–2^	*g*_1_ (eV)	*g*_2_ (eV)	n_medium
mode 1	2.60	0.00095	0.100	0.060	1.8
mode 2	1.87	0.00120	0	0	1.8
mode 3	3.40	0.00050	0	0	2.0

**Table 3:** P-pol

cavity mode	*E*_c0_ (eV)	α(eV·μm^2^) α ×10^–2^	*g*_1_ (eV)	*g*_2_(eV)	n_medium
mode 1	2.60	0.11	0.100	0.060	1.8
mode 2	1.87	0.15	0	0	1.8
mode 3	3.40	0.050	0	0	2.0

The Rabi splitting is evaluated at the point where the cavity mode and the exciton have the same energy. In our system, due to the perovskite layer’s refractive index and thickness, this resonance occurs at a finite in-plane momentum of *k* = 20 μm^–1^.

#### 4.4.8 Nuclear Magnetic Resonance

NMR spectra were acquired on a Bruker Avance III 600 MHz (600.13 MHz) spectrometer, fit with a 5 mm QCI cryoprobe. The temperature was actively monitored at 298 K, the matching, tuning resolution and 90° were automatically adjusted.

^1^H NMR spectra were performed with 32 scans without steady ones, with interpulses delay of 30 s, 65,536-digit points, over a spectral width of 20.83 ppm with the offset positioned at 6.18 ppm. Spectra were smoothed with an exponential function equivalent to 0.3 Hz prior to the Fourier transform.

^13^C NMR spectra were acquired with a 30° for the ^13^C excitation pulse, with 18435 transients, a relaxation delay of 2 s, over a spectral width of 236.65 ppm centered at 100.00 ppm.

#### 4.4.9 Atomic Force Microscopy

AFM images were acquired in tapping mode with standard Si cantilevers (PPP-NCHR) mounted on a Park X-100 AFM.

#### 4.4.10 Atomistic Simulations

All atomistic simulations are performed within the framework of density functional theory (DFT) using the Vienna Ab initio simulation package (VASP).^63,65^ The interaction between valence electrons and ionic cores is described using the projector augmented-wave (PAW) method.^64^ The plane-wave basis set is expanded up to a kinetic energy cutoff of 500 eV. Brillouin-zone integrations were carried out using a Γ-centered k-point mesh with a resolution of 0.30/2π Å^–1^. Two types of exchange correlation-functional are adopted: the Perdew–Burke–Ernzerhof (PBE)^61^ and the more accurate and more computationally demanding Heyd-Scuseria-Ernzerhof (HSE06).^87^ The approach is the following. First, band structure, density of states (DOS), and partial charge densities are calculated with PBE, neglecting spin–orbit coupling (SOC). This is generally known to provide, in lead halide perovskites, an error compensation in band gap (and likely in the energy difference among electronic states in general) between the neglection of SOC, which produces an overestimation of the band gap, and the adoption of a GGA functional such as PBE, which tends to underestimate the band gap. Finally, the HSE06 functional is adopted to have an accurate estimation of the electronic energy levels alignment, without relying on error compensation (Figure S15). The geometry is kept fixed at the experimental estimate. Electronic self-consistency is achieved with an energy convergence threshold of 5 × 10^–7^.

## Supplementary Material



## Notes

Additional data, including NMR analysis, XRD analysis, XPS, SEM elemental mapping (EDS), XPS elemental analysis, optical characterization, AFM, UPS analysis, DFT analysis, and PD characterization, are presented (PDF)
