# Supplementary material for: π‑Conjugated Indole-Based 2D Perovskite Microcavities for Self-Powered Ultraviolet Photodetection
Source: Chem Mater. 2026 Jul 22;38(15):7864–77. doi: 10.1021/acs.chemmater.6c01061 (PMC13471732; doi:10.1021/acs.chemmater.6c01061)
Supplement: Supplementary file 1 [file cm6c01061_si_001.pdf]

Supporting Information

π-Conjugated Indole-Based 2D Perovskite Microcavities for Self-Powered Ultraviolet Photodetection

Meenakshi Pegu[1], Mukesh K. Thakur[2], Sudhir K. Saini[3], Aarya Prabhakaran[1], Gabriele Saleh[1], Mehmet Z. Akgul[1], Shence Zhang[1,7], Francesco De Boni[4], Sergio Marras[4], Simone Lauciello[5], Luca Goldoni[4], Matteo Lorenzoni[4], Serena De Negri[6], Pavlo Solokha[6], Roman Krahne[3]*, Liberato Manna[1]*

[1] Nanochemistry, Istituto Italiano di Tecnologia, Via Morego 30, 16163 Genova, Italy.

[2] Photonic Nanomaterials, Istituto Italiano di Tecnologia, Via Morego 30, 16163 Genova, Italy.

[3] Optoelectronics Research Line, Istituto Italiano di Tecnologia, Via Morego 30, 16163 Genova, Italy.

[4]Materials Characterization Facility, Istituto Italiano di Tecnologia, Via Morego 30, 16163 Genova, Italy.

[5]Electron Microscopy, Istituto Italiano di Tecnologia, Via Morego 30, 16163 Genova, Italy.

[6] Dipartimento di Chimica e Chimica Industriale, Università degli Studi di Genova, Via Dodecaneso 31, Genova, 16146, Italy.

[7]Department of Chemistry, Materials and Chemical Engineering, Politecnico di Milano, Piazza Leonardo da Vinci 32, Milano 20133, Italy.

AUTHOR INFORMATION

Corresponding Author

roman.krahne@iit.it

liberato.manna@iit.it

Supplementary Data Figures and Tables:

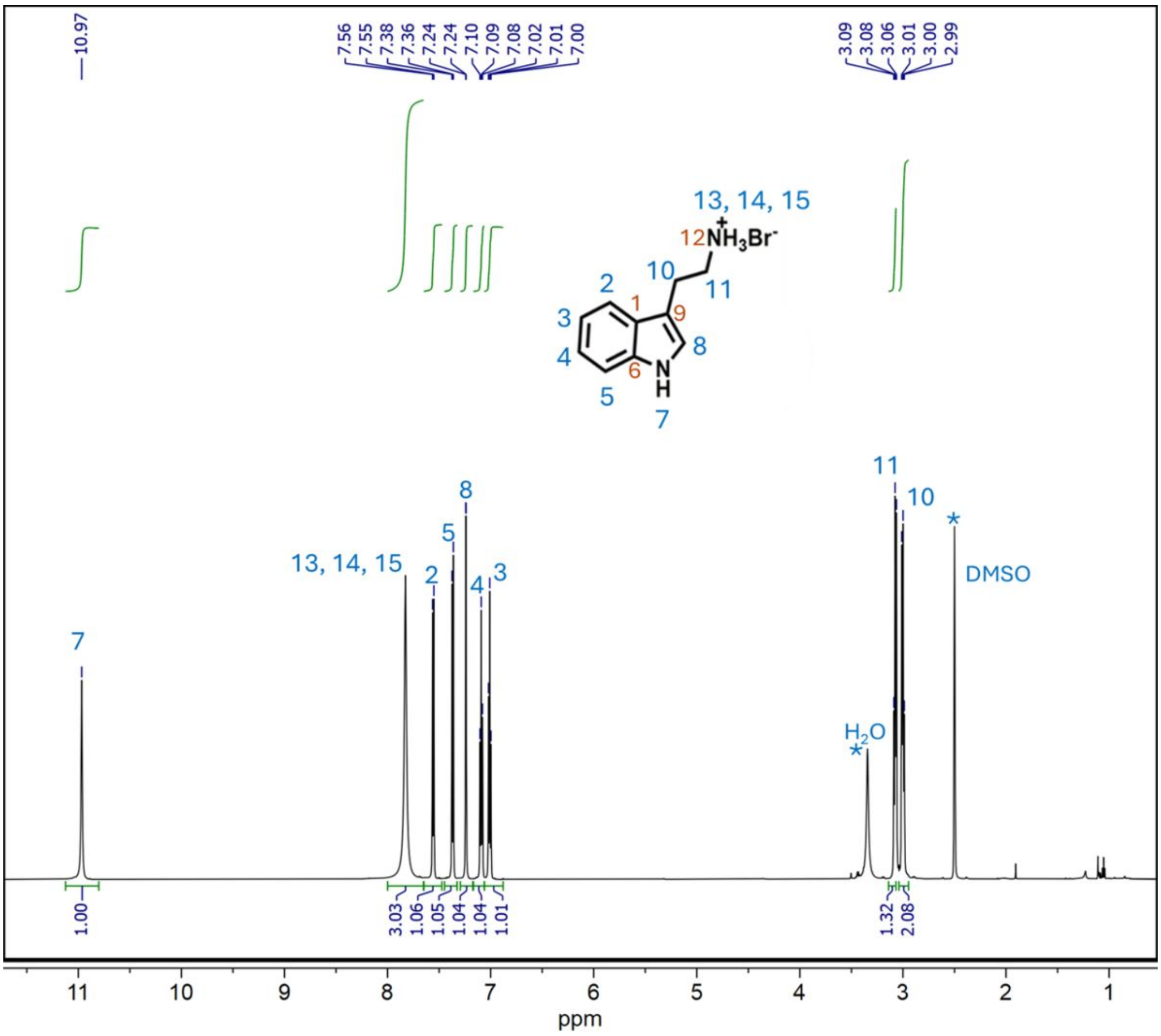

Figure S1: ^{1}H NMR spectrum of the TPA-Br salt in DMSO-d6.

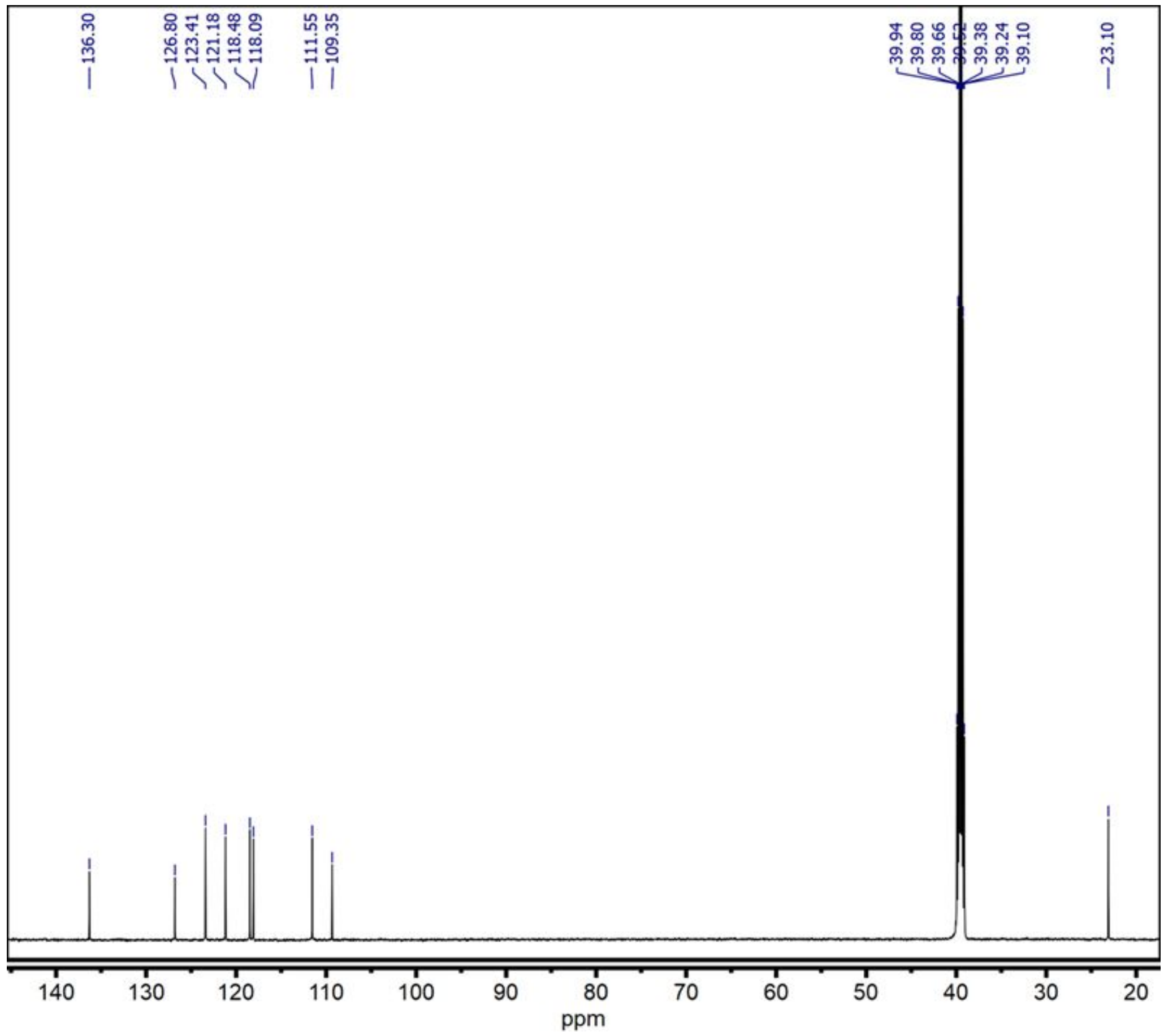

Figure S2: ^{13}C NMR spectrum of the TPA-Br salt in DMSO-d6.

Table S1. Selected crystallographic data and structure refinement parameters for the TPA-Br single crystals.

**Compound**	**TPA-Br**
**CCDC number**	2478831
**Empirical formula**	$C_{10}H_{13}BrN_2$
**Formula weight**	241.13
**Crystal system**	orthorhombic
**Space group (number), Z**	$Pbca$ (61), 8
**a [Å]**	8.551(2)
**b [Å]**	10.368(2)
**c [Å]**	24.259(6)
**Volume [Å^3]**	2150.6(9)
**Calculated density, ρ_{calc} [g/cm^3]**	1.489
**Absorption coefficient, μ [mm^{-1}]**	3.78
**F(000)**	976
**2θ range [°]**	5.83 to 47.73 (0.88 Å)
**Index ranges**	$-8 \le h \le 9$ $-11 \le k \le 10$ $-27 \le l \le 27$
**Diffraction measurement method**	1 ω scan, 1 φ scan
**Reflections collected**	7182
**Independent reflections**	1578 R_{int} = 0.1476 R_{sigma} = 0.1354
**Coverage of independent reflections**	94.9
**Data/Restraints/Parameters**	1578/0/119
**Goodness-of-fit on F^2**	1.011
**Final R indexes [I≥2σ(I)]**	R_1 = 0.0613 wR_2 = 0.0977
**Final R indexes [all data]**	R_1 = 0.1615 wR_2 = 0.1313
**Largest peak/hole [eÅ^{-3}]**	0.34/−0.35

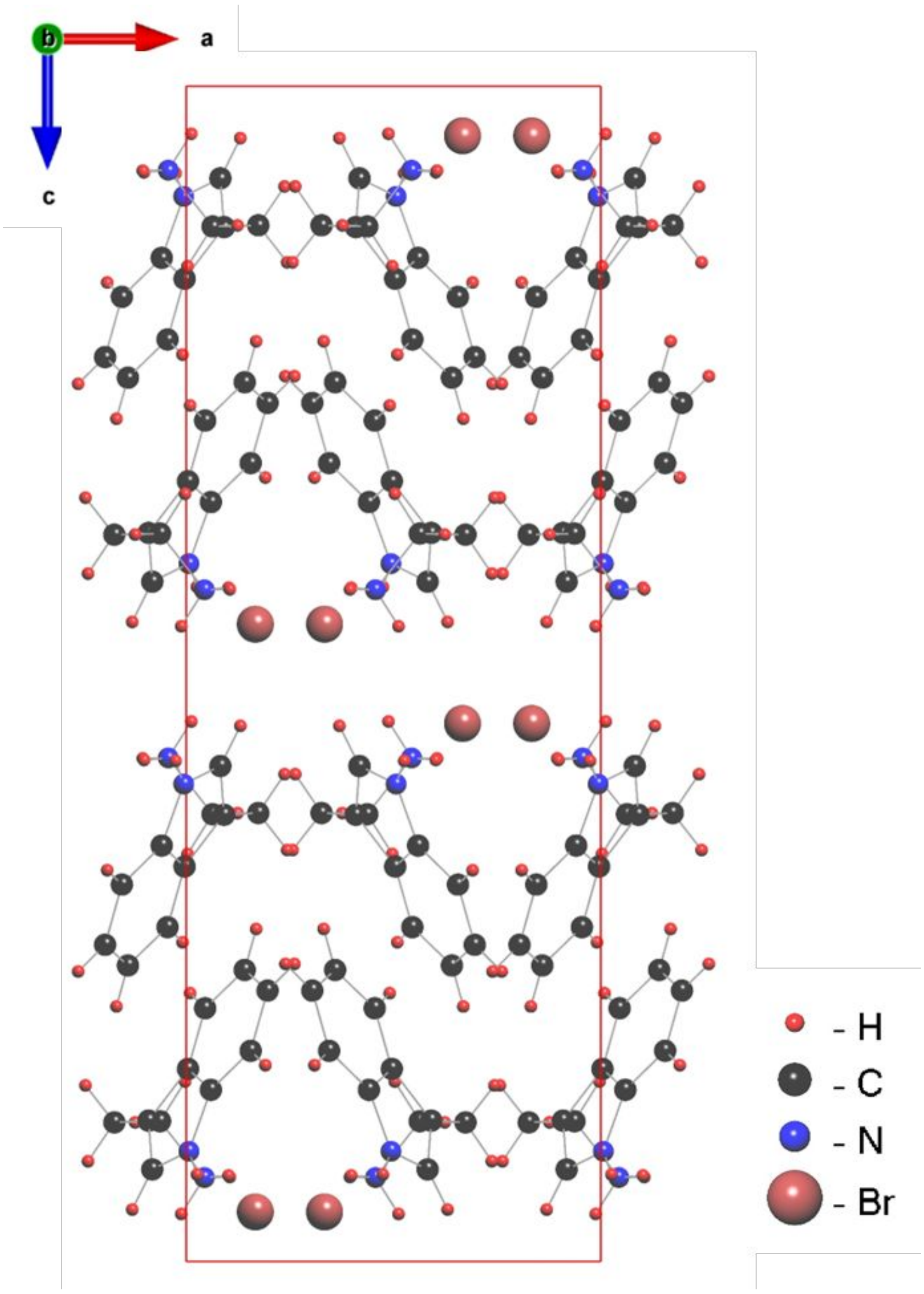

Figure S3: Single Crystal structure of the tryptammonium bromide (TPA-Br) salt. Carbons (black), hydrogens (light grey), nitrogen (blue), and bromine (yellow).

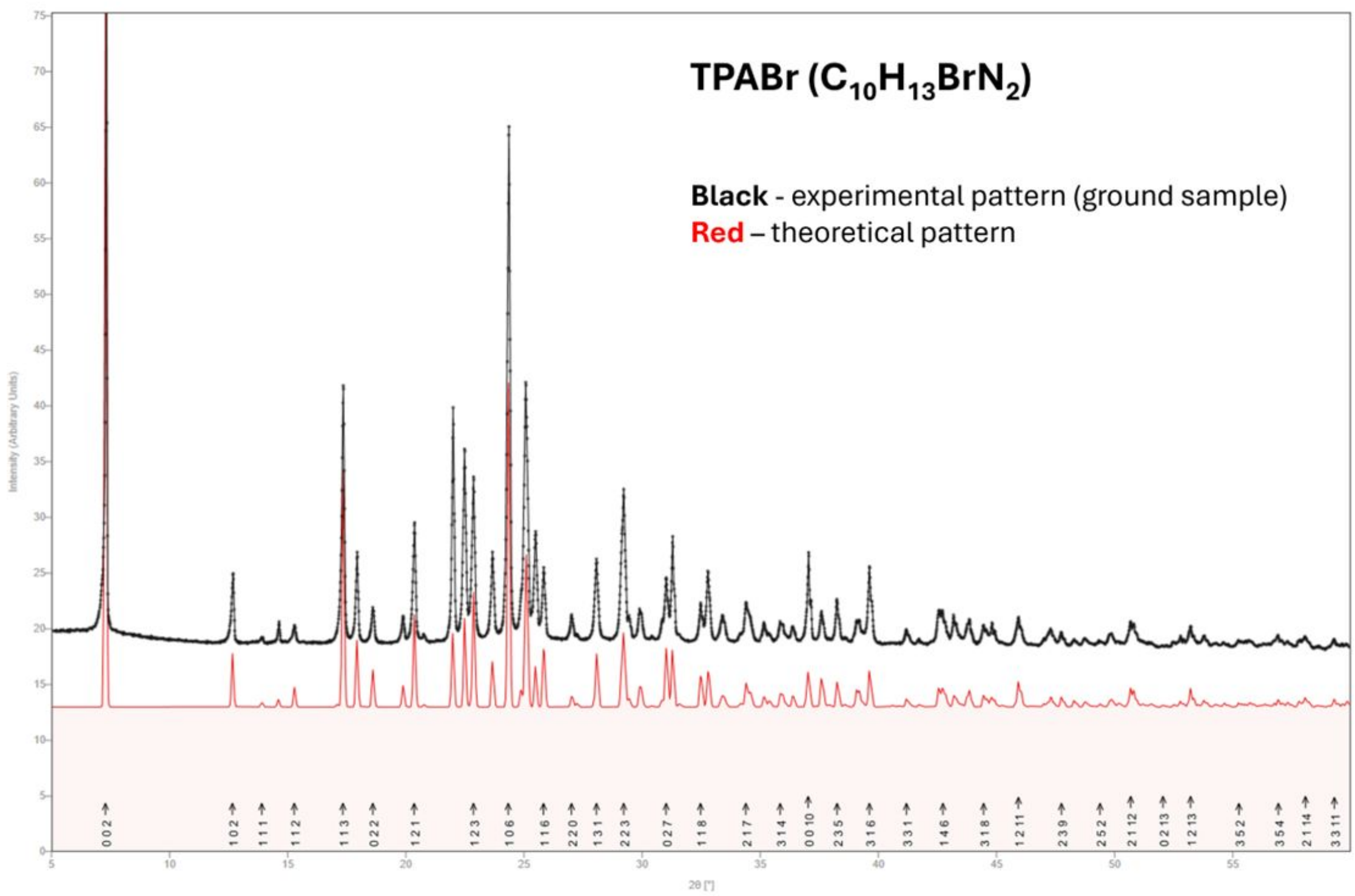

Figure S4: X-ray diffraction (XRD) pattern of the TPA-Br salt.

Table S2. Atomic coordinates and Ueq [Å^2] for TPA-Br

Atom	x/a	y/b	z/c	U_{eq}
**Br01**	0.33348(12)	0.14851(8)	0.45776(4)	0.0632(4)
**N002**	0.5435(8)	0.1445(6)	0.5715(3)	0.055(2)
**H00G**	0.605054	0.075313	0.571914	0.082
**H00H**	0.601976	0.215436	0.573293	0.082
**H00I**	0.487883	0.145375	0.540498	0.082
**N003**	0.5063(9)	0.5733(7)	0.5938(3)	0.055(2)
**H003**	0.525786	0.640221	0.574030	0.066
**C004**	0.5593(11)	0.5519(9)	0.6461(4)	0.044(2)
**C005**	0.5041(11)	0.4331(8)	0.6639(4)	0.043(2)
**C006**	0.4178(11)	0.4708(9)	0.5784(4)	0.057(3)
**H006**	0.369534	0.462304	0.544204	0.069
**C007**	0.4366(11)	0.1404(9)	0.6194(4)	0.057(3)
**H00A**	0.496996	0.141362	0.653197	0.068
**H00B**	0.376435	0.061061	0.618444	0.068
**C008**	0.5454(11)	0.3908(8)	0.7155(4)	0.051(3)
**H008**	0.509181	0.311809	0.728380	0.061
**C009**	0.6409(12)	0.4663(10)	0.7481(4)	0.064(3)
**H009**	0.668505	0.436842	0.782970	0.076
**C00A**	0.4091(11)	0.3817(8)	0.6199(4)	0.051(3)
**C00B**	0.3263(11)	0.2552(8)	0.6184(4)	0.058(3)
**H00C**	0.262877	0.251003	0.585328	0.070
**H00D**	0.256562	0.249729	0.649881	0.070
**C00C**	0.6554(12)	0.6285(8)	0.6793(5)	0.061(3)
**H00E**	0.690847	0.708302	0.667027	0.073
**C00D**	0.6960(13)	0.5832(10)	0.7305(5)	0.070(3)
**H00F**	0.760931	0.631762	0.753249	0.084

Table S3. Atomic coordinates and Ueq [Å^2] for TPA_2PbBr_4

Atom	x/a	y/b	z/c	U_{eq}
**Pb1_1**	1.000000	0.500000	0.500000	0.03683(10)
**Br1_1**	0.80751(8)	0.19138(8)	0.50873(2)	0.0539(2)
**Br2_1**	0.93846(8)	0.49162(8)	0.42517(2)	0.0594(2)
**N1_2**	0.6896(7)	0.3671(7)	0.32437(15)	0.0745(18)
**H1_2**	0.775460	0.320798	0.316614	0.089
**N2_2**	0.5427(7)	0.5200(7)	0.43458(15)	0.097(3)
**H2A_2**	0.560792	0.604103	0.448590	0.145
**H2B_2**	0.627879	0.506073	0.421076	0.145
**H2C_2**	0.526358	0.428742	0.446652	0.145
**C1_2**	0.4786(7)	0.5340(7)	0.33034(16)	0.0465(17)
**C2_2**	0.6069(8)	0.4931(8)	0.30909(17)	0.0554(17)
**C3_2**	0.6377(9)	0.5726(9)	0.27850(17)	0.066(2)
**H3_2**	0.724564	0.543345	0.264803	0.079
**C4_2**	0.5334(10)	0.6966(10)	0.26950(19)	0.080(2)
**H4_2**	0.548913	0.751756	0.249023	0.096
**C5_2**	0.4062(9)	0.7412(10)	0.2902(2)	0.082(2)
**H5_2**	0.338306	0.826514	0.283475	0.099
**C6_2**	0.3779(8)	0.6616(9)	0.32058(18)	0.065(2)
**H6_2**	0.292094	0.693267	0.334369	0.078
**C7_2**	0.4847(7)	0.4245(8)	0.35927(16)	0.0477(16)
**C8_2**	0.6127(8)	0.3270(8)	0.35409(18)	0.065(2)
**H8_2**	0.645115	0.243256	0.368709	0.078
**C9_2**	0.3690(7)	0.4241(8)	0.38807(15)	0.0541(18)
**H9A_2**	0.373210	0.317334	0.399134	0.065
**H9B_2**	0.260685	0.438553	0.379174	0.065
**C10_2**	0.4003(7)	0.5545(8)	0.41395(17)	0.0563(19)
**H10A_2**	0.414795	0.659277	0.402495	0.068
**H10B_2**	0.306994	0.564033	0.428718	0.068

U_{eq} is defined as 1/3 of the trace of the orthogonalized U_{ij} tensor.

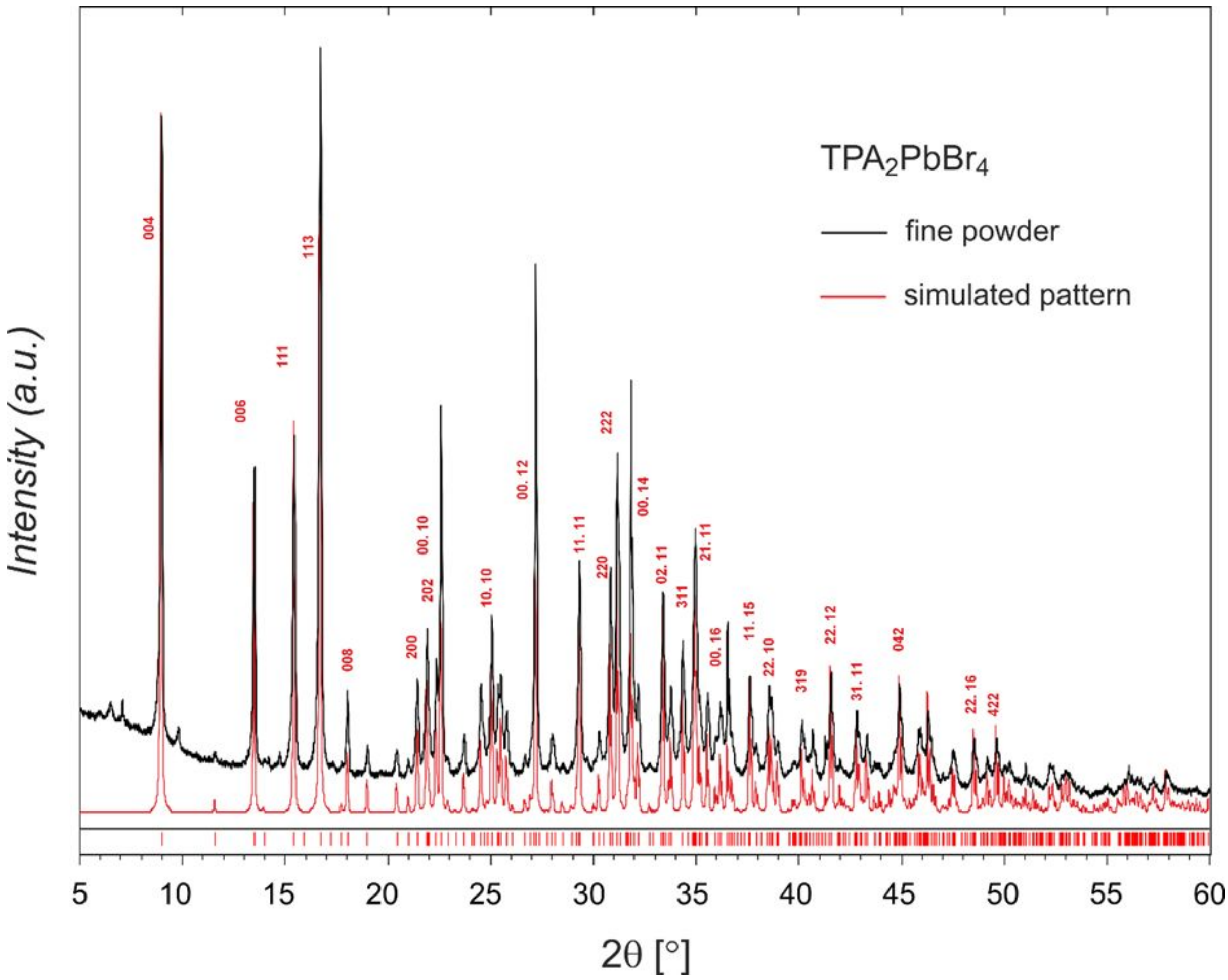

Figure S5: p-XRD pattern of the TPA_2PbBr_4 of fine powder, matching the corresponding simulated pattern

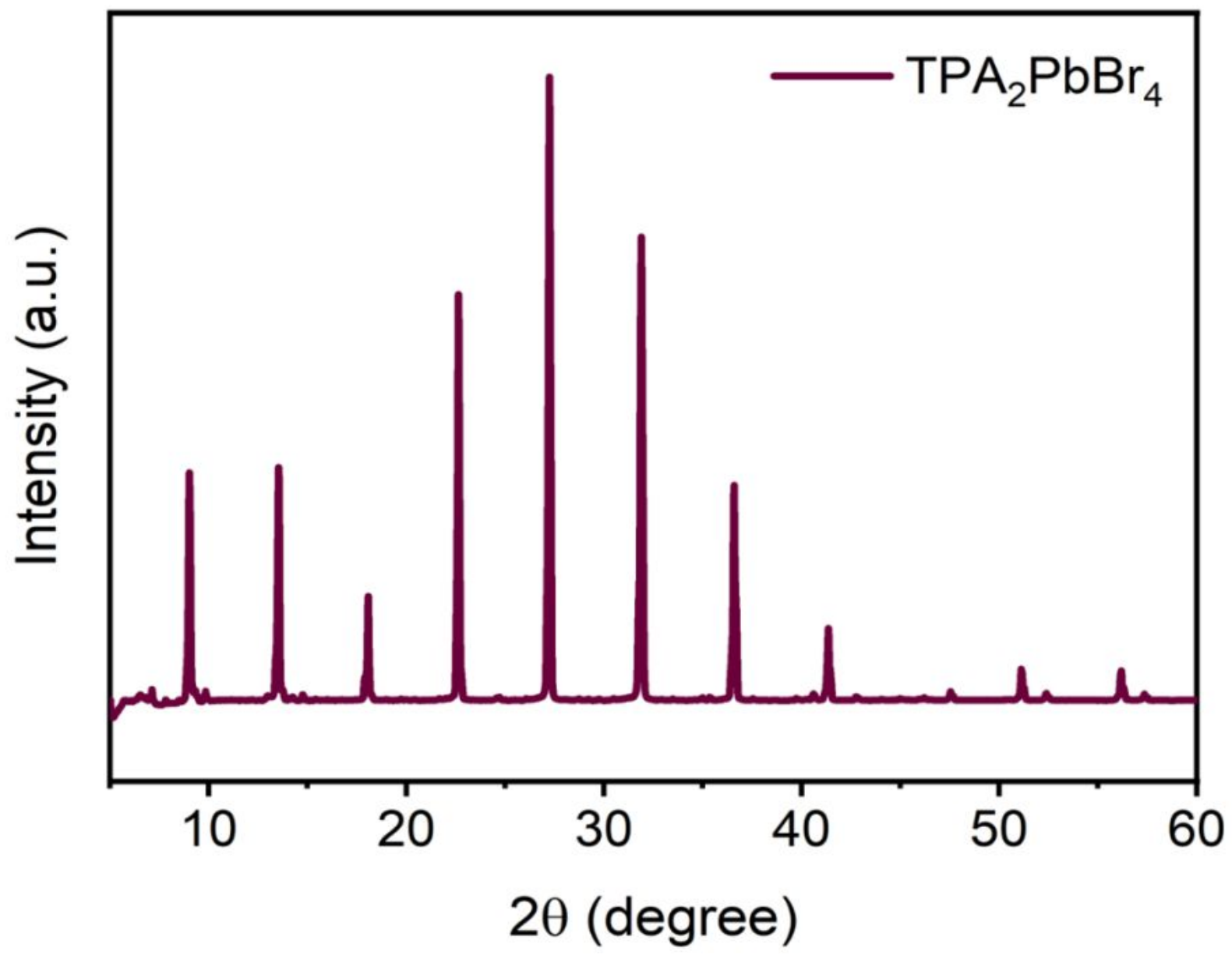

Figure S6: XRD pattern of the TPA_2PbBr_4 perovskite after six months of storage at ambient conditions.

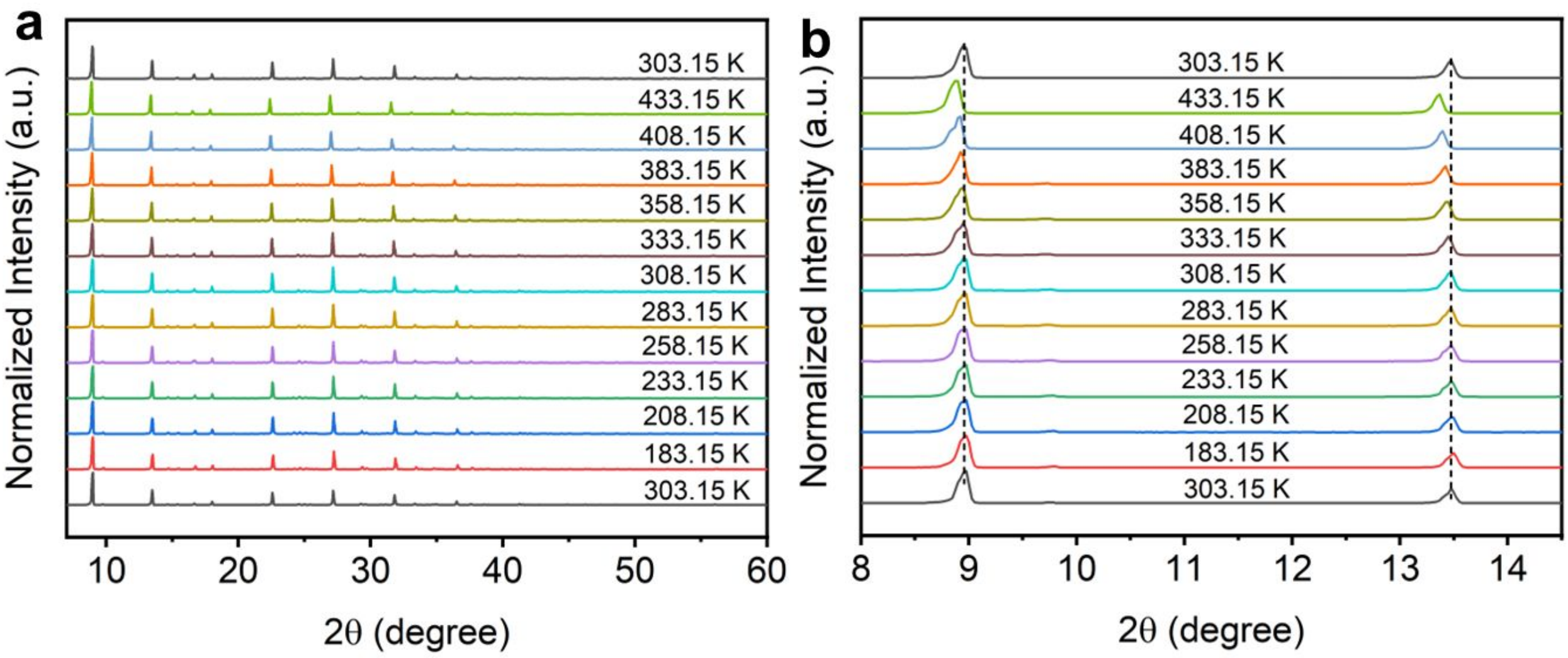

Figure S7: (a-b) Temperature-dependent XRD analysis of the TPA_2PbBr_4 microflakes recorded from 183 K to 433 K. XRD analysis at 303 K is performed before and after 183K and 433 K.

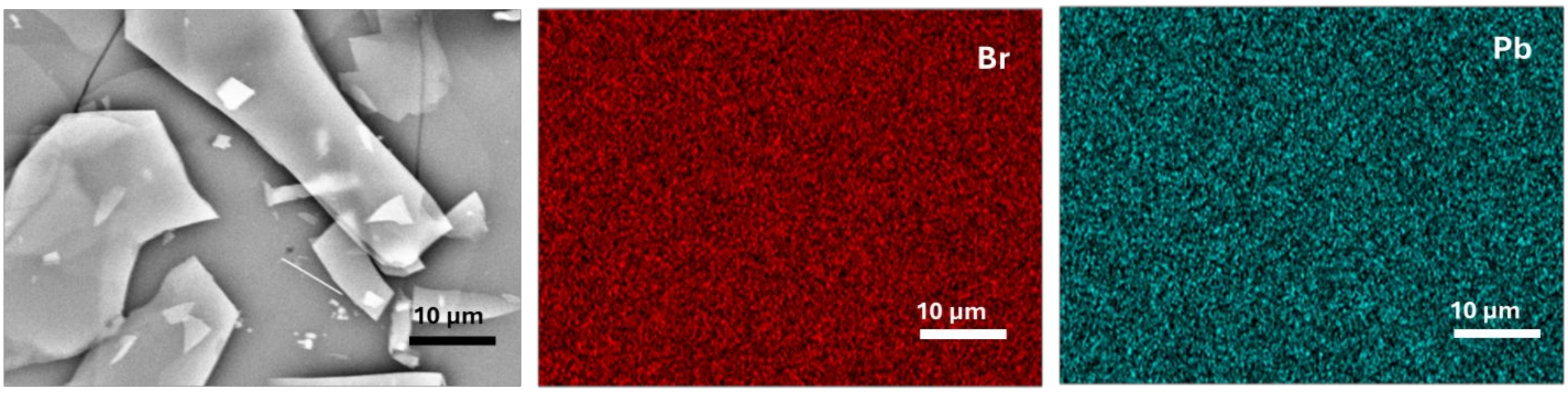

Figure S8: SEM-EDS of the TPA_2PbBr_4 perovskite microflakes.

Table S4: Elemental composition of the TPA_2PbBr_4 microflakes.

**Map Sum Spectrum**	**Line Type**	**Wt%**	**Wt% Sigma**	**Atomic %**
**Br**	K series	58.60	0.19	78.59
**Pb**	L series	41.40	0.19	21.41
**Total**		100.00		100.00

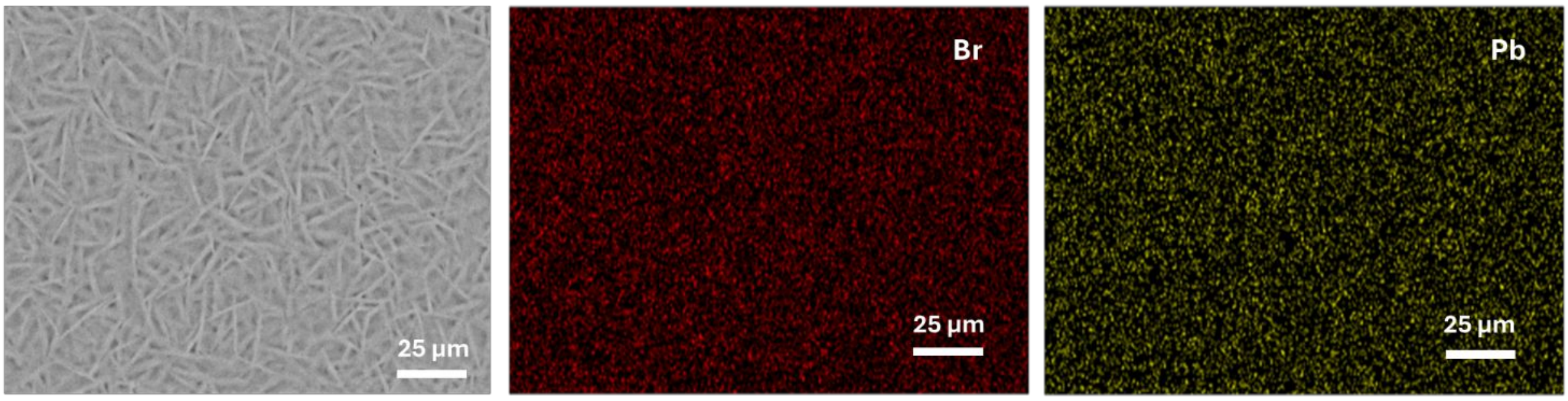

Figure S9: SEM-EDS of the TPA_2PbBr_4 perovskite film.

Table S5: Elemental composition of the TPA_2PbBr_4 thin film.

**Map Sum Spectrum**	**Line Type**	**Wt%**	**Wt% Sigma**	**Atomic %**
**Br**	K series	60.53	0.60	79.90
**Pb**	L series	39.47	0.60	20.10
**Total**		100.00		100.00

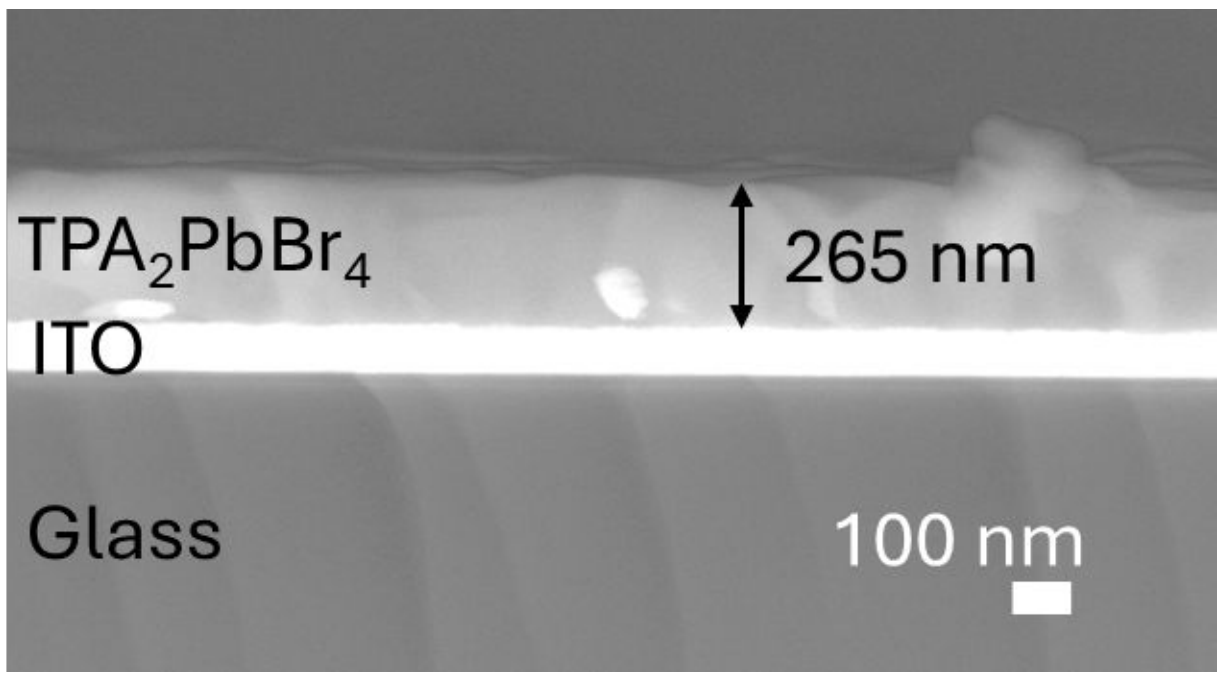

Figure S10: Cross-sectional SEM image of the TPA_2PbBr_4 perovskite thin film.

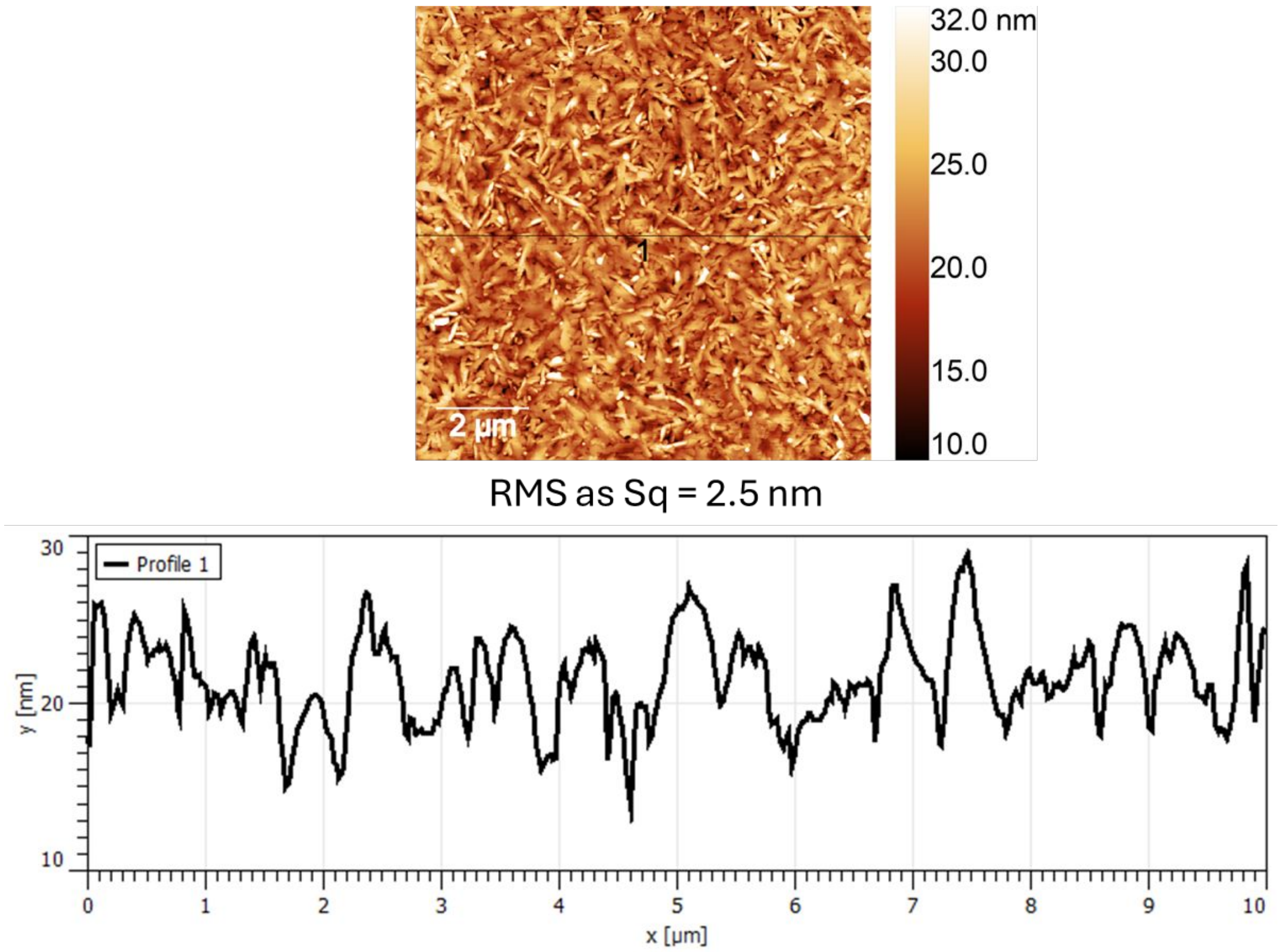

Figure S11: AFM surface height profile of the TPA_2PbBr_4 perovskite thin film.

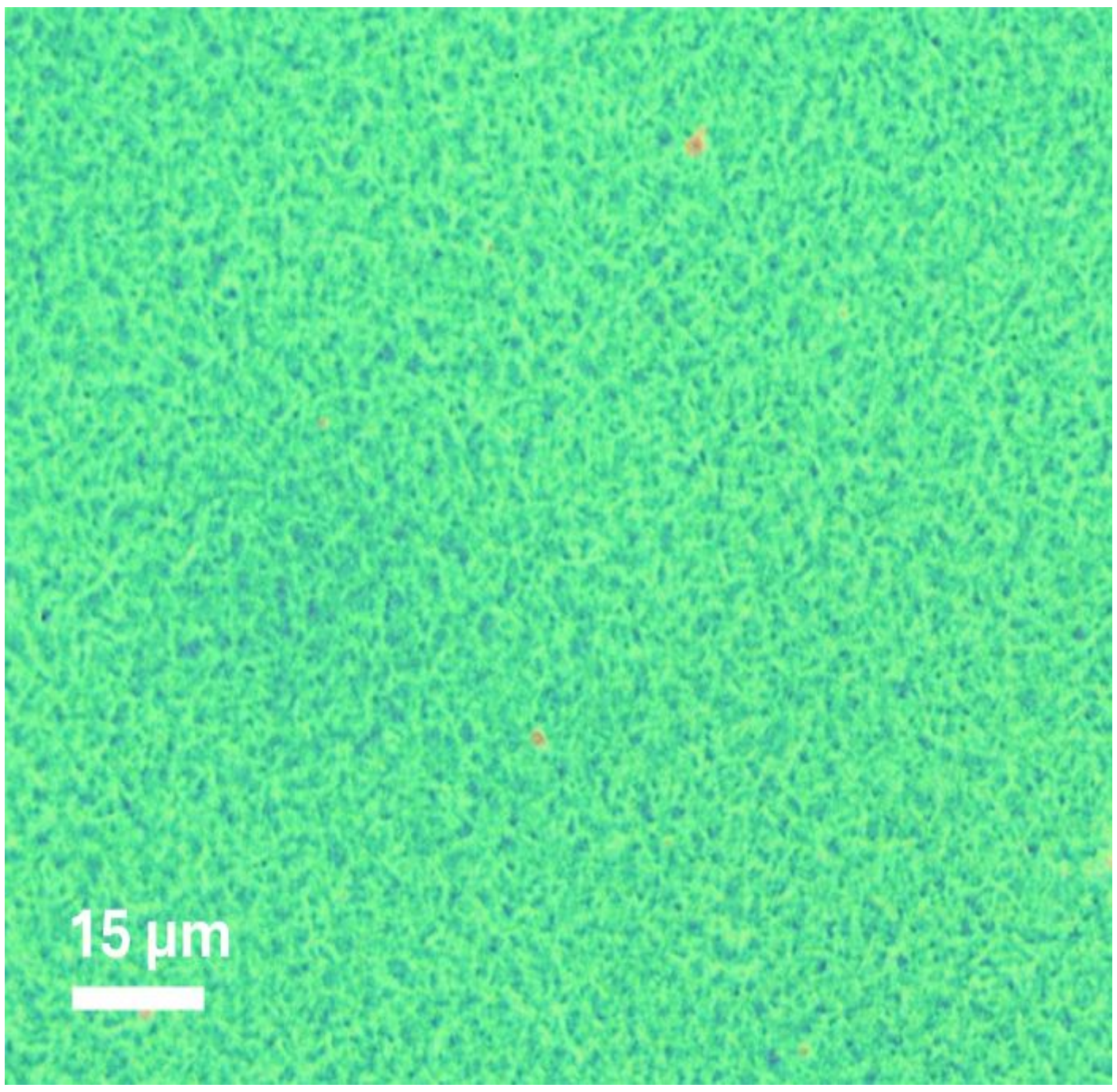

Figure S12: Optical micrograph of a TPA_2PbBr_4 layered perovskite thin film.

Table S6: Compositional analysis of the TPA_2PbBr_4 by XPS analysis.

Sample	% N	% Pb	% Br	Br/Pb
**TPA_2PbBr_4**	47.0	12.5	40.5	3.24

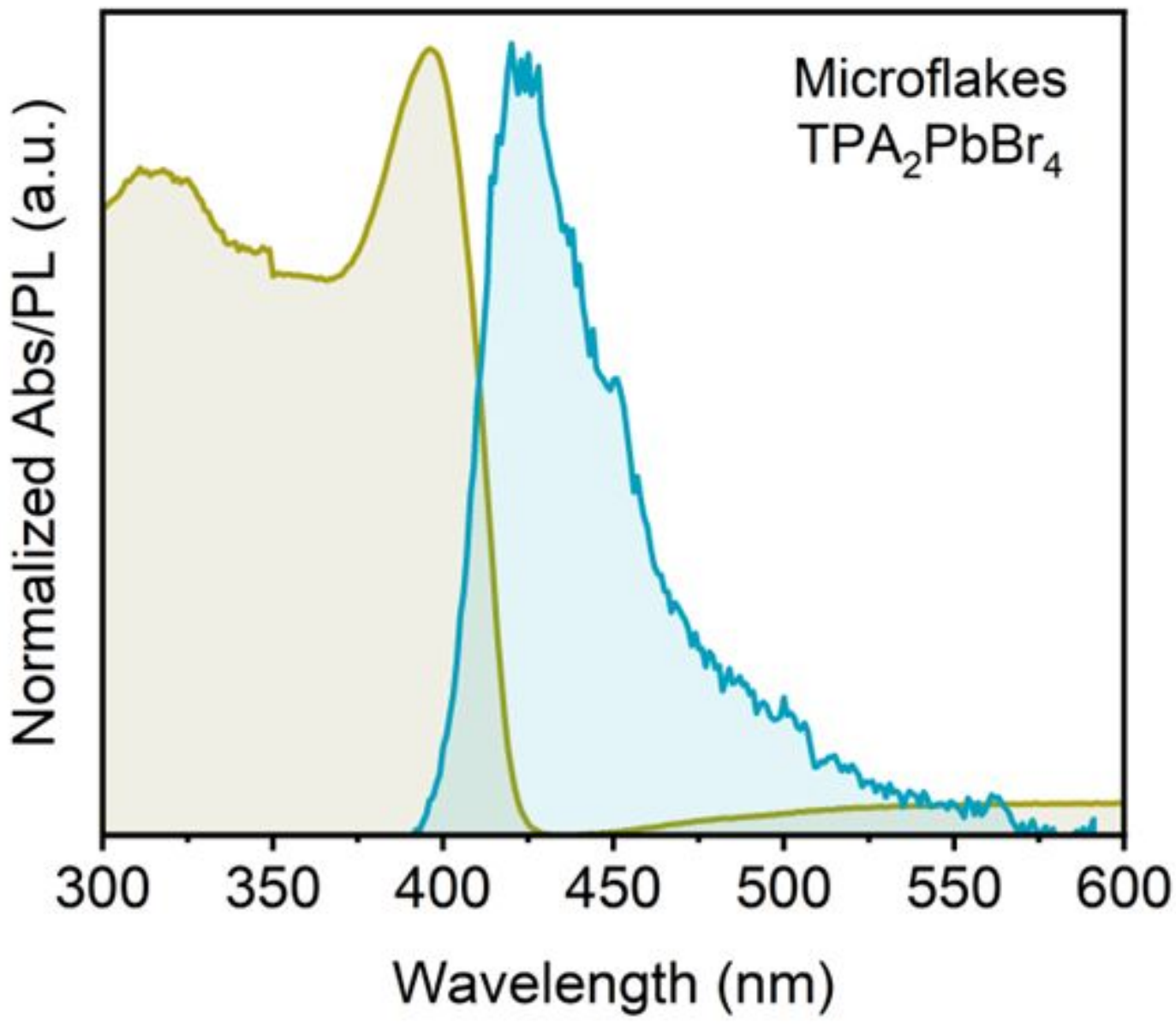

Figure S13: UV-Visible and photoluminescence spectra of theTPA_2PbBr_4 perovskite microflakes.

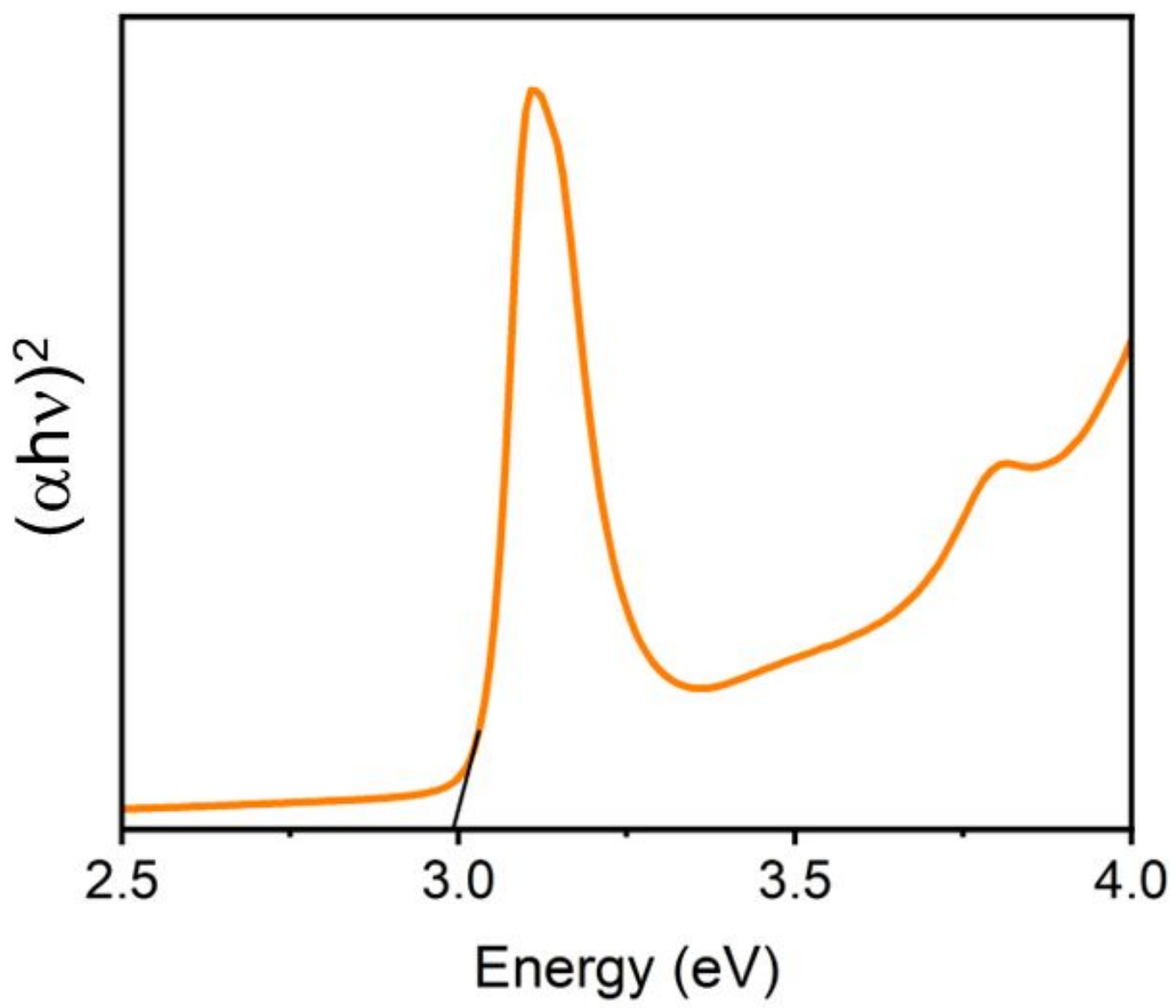

Figure S14: Tauc plot of the layered TPA_2PbBr_4 perovskite thin film.

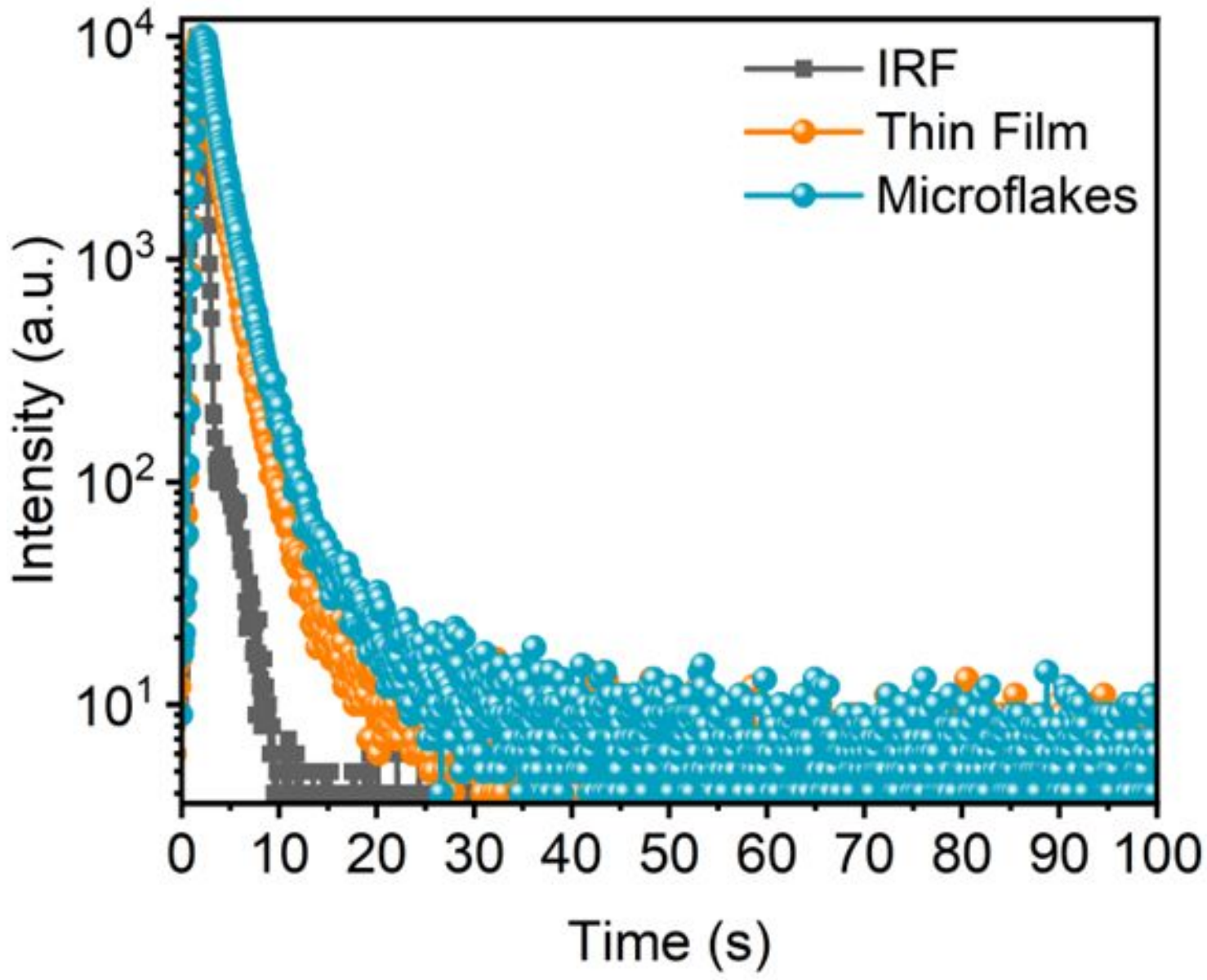

Figure S15: PL decay of the TPA_2PbBr_4 perovskite thin film and microflakes.

Table S7: PL Lifetime parameters

TPA_2PbBr_4	A_1	τ_1 (ns)	A_2	τ_2 (ns)	R_2	τ_{ave} (ns)
Microflakes	5843.40137	1.9303	4153.60976	4.03226	0.99999	3.2
Thin film	7103.99841	1.37939	2819.18748	3.08619	0.99999	2.18

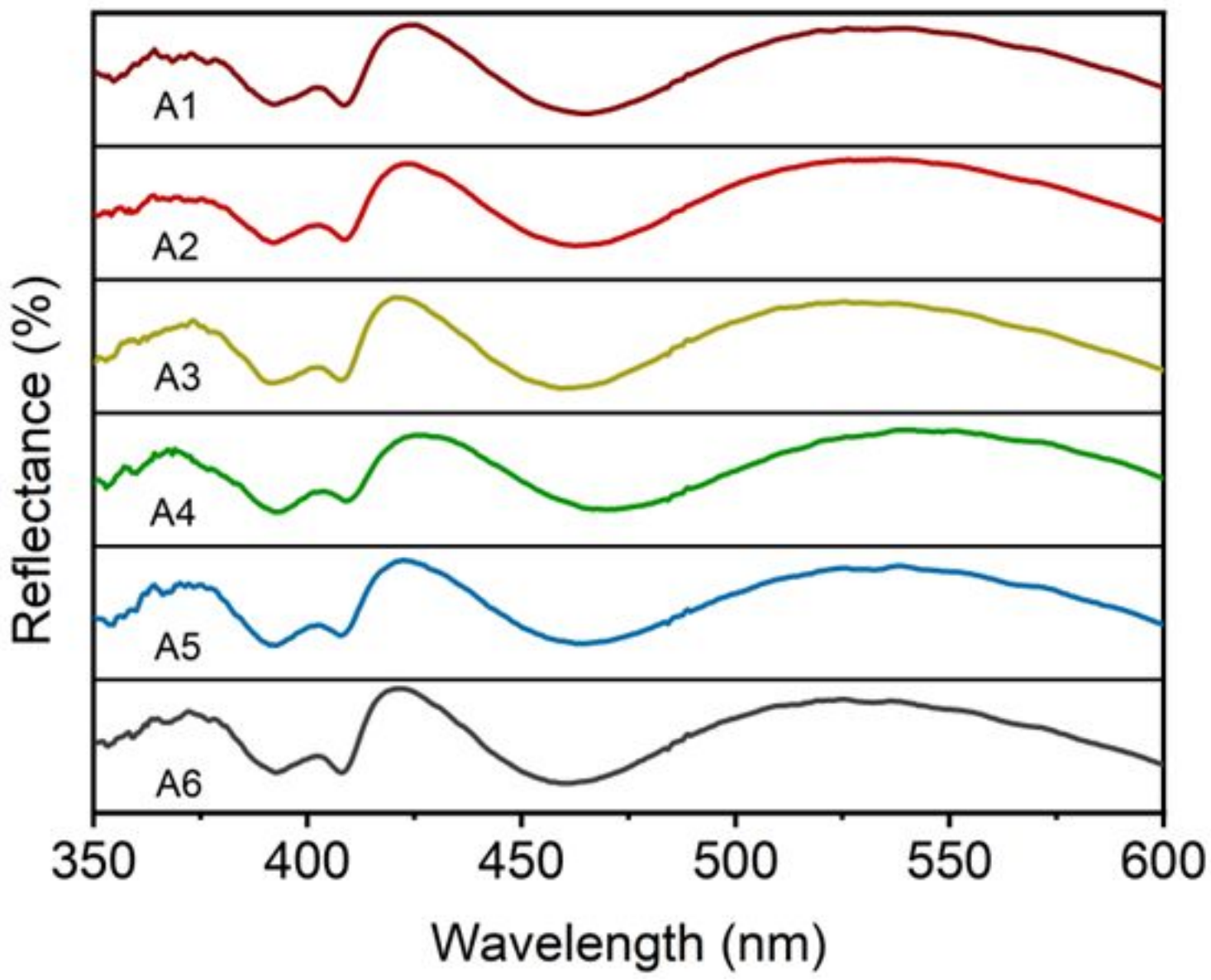

Figure S16: Reflectance under normal incidence with a series of minima that can be associated with the confined cavity modes, measured at six different positions of the same homogeneous TPA_2PbBr_4 thin film (A = positions).

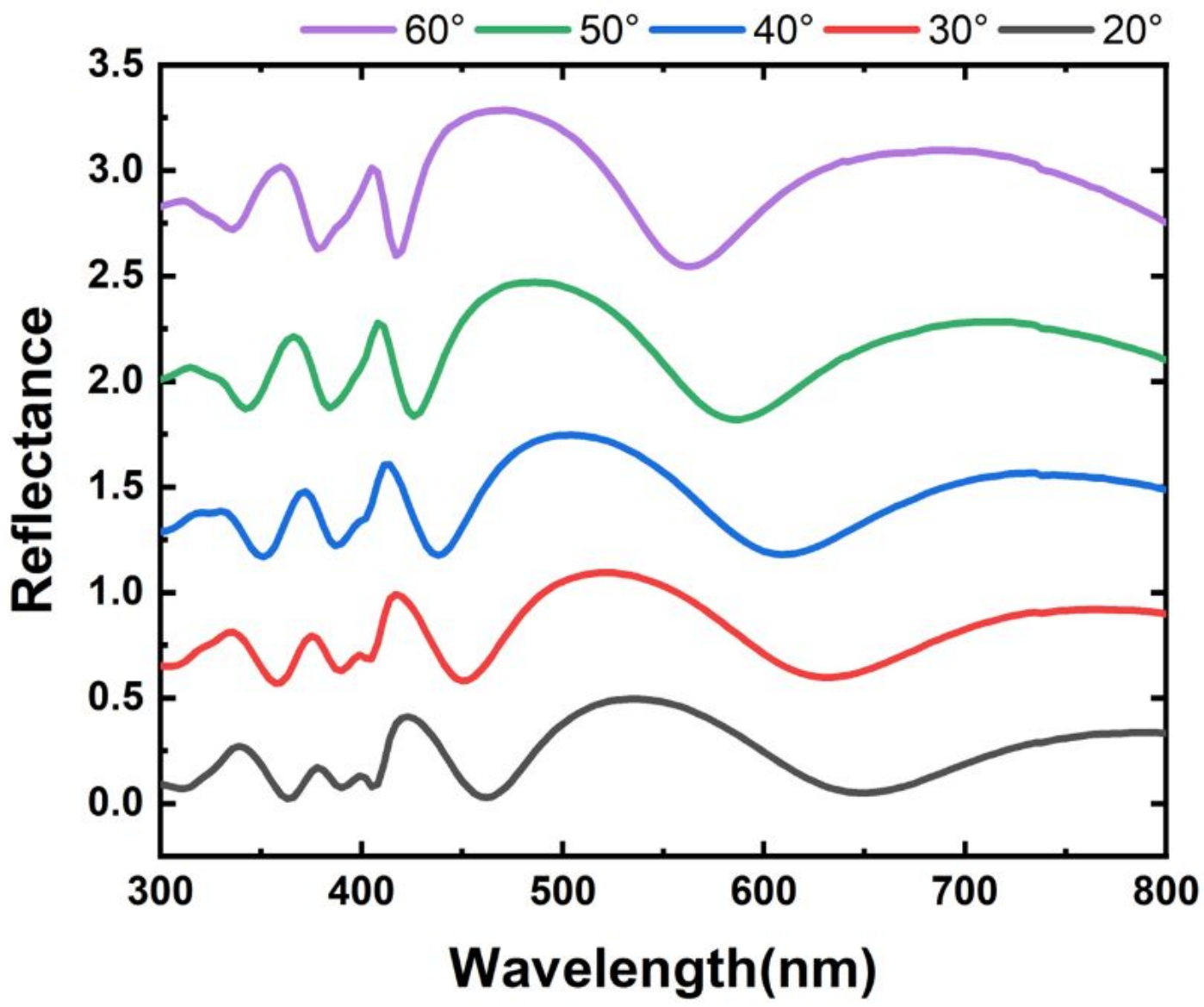

Figure S17: Reflectance under s-polarised light at different angles with a series of minima that can be associated with the confined cavity modes.

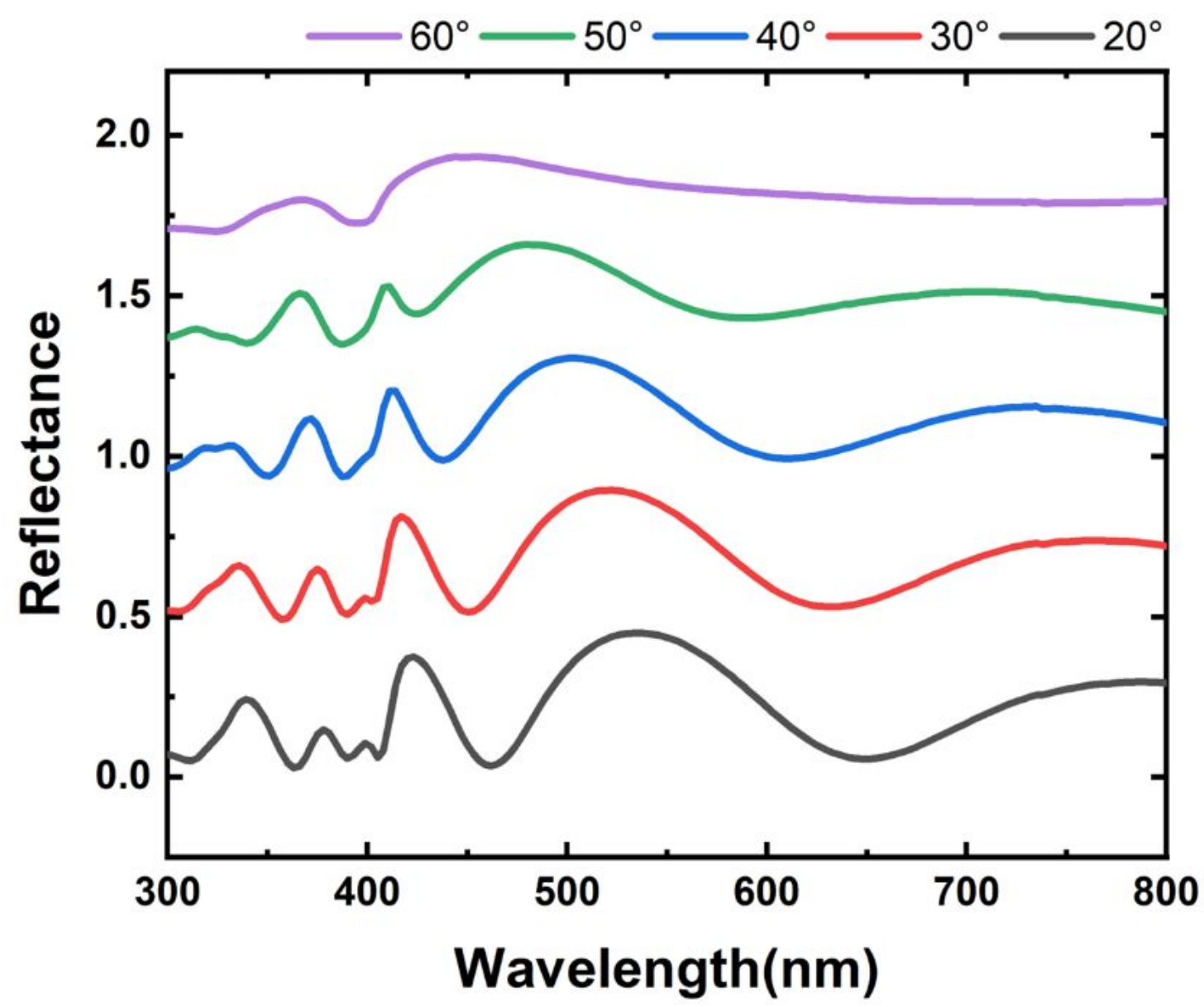

Figure S18: Reflectance under p-polarized light at different angles with a series of minima that can be associated with the confined cavity modes.

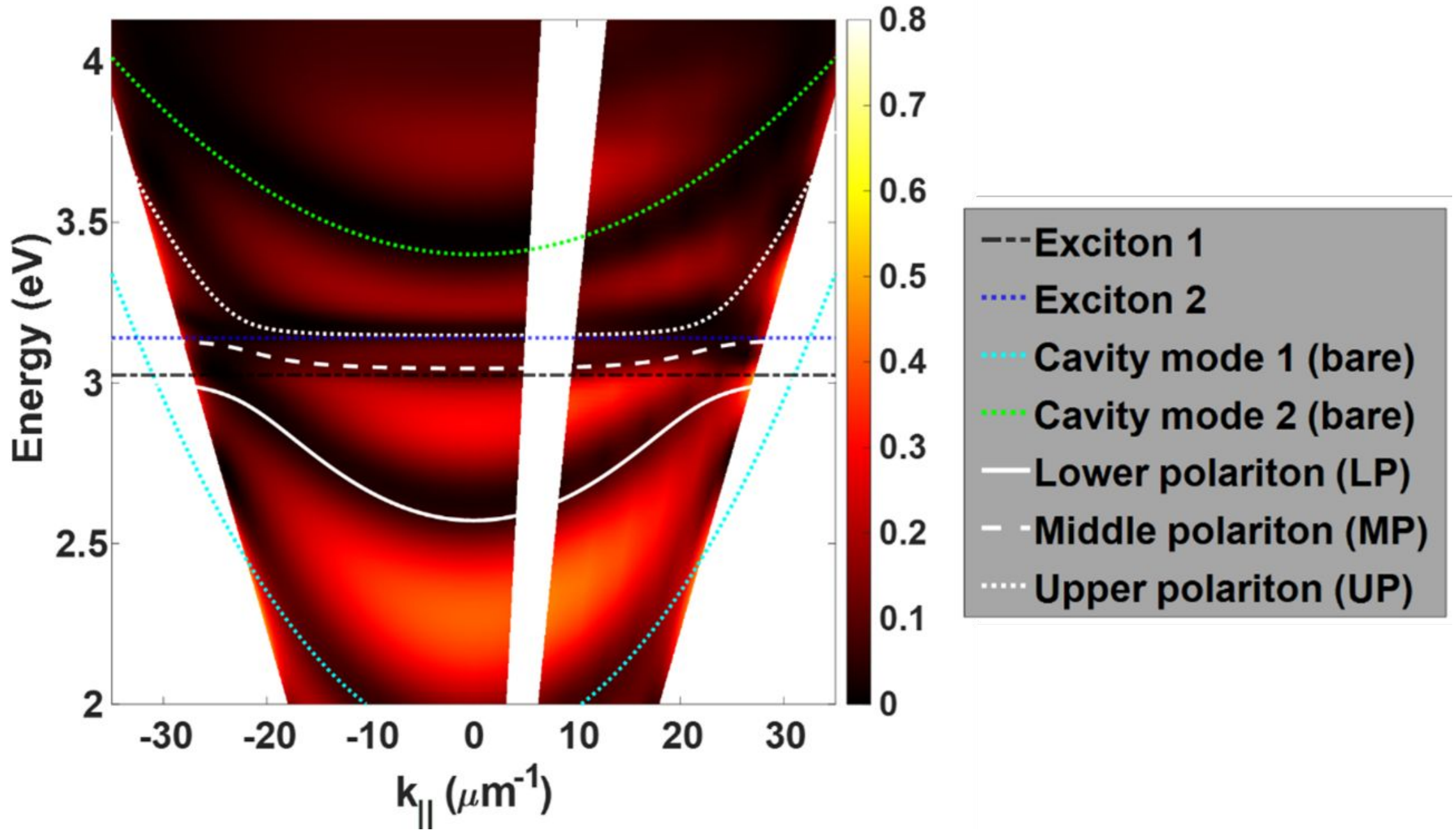

Figure S19: Color-plot of the calculated (left) and measured (right) of p-polarised reflectance intensity versus in-plane wave vector and energy of the incident light.

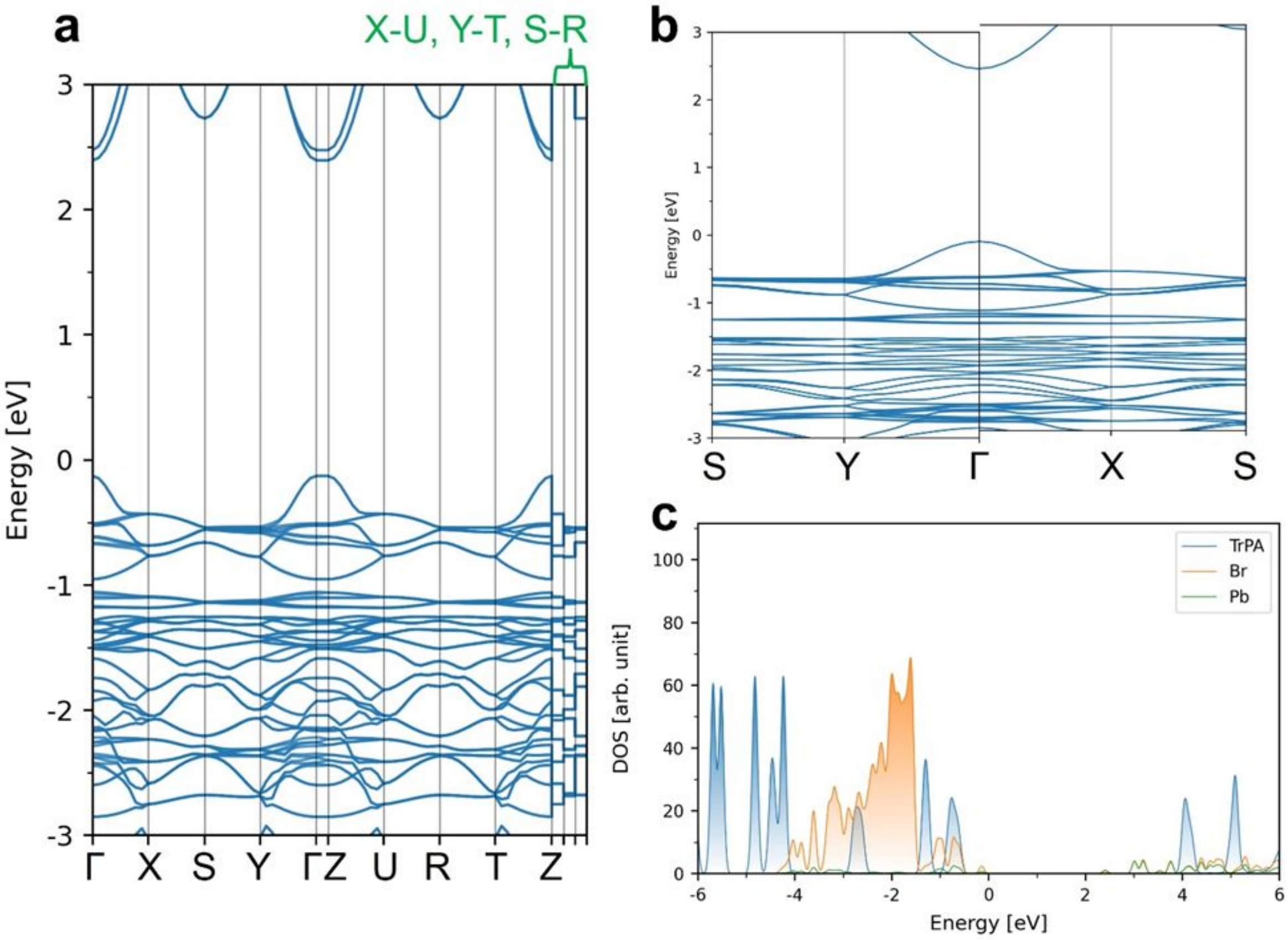

Figure S20. Band dispersion diagrams and density of states (DOS) of the TPA_2PbBr_4. The PBE and HSE06+SOC band diagrams (see Methods section of the main text) are shown in panels a and b, respectively, while panel c reports the DOS obtained with HSE06+SOC.

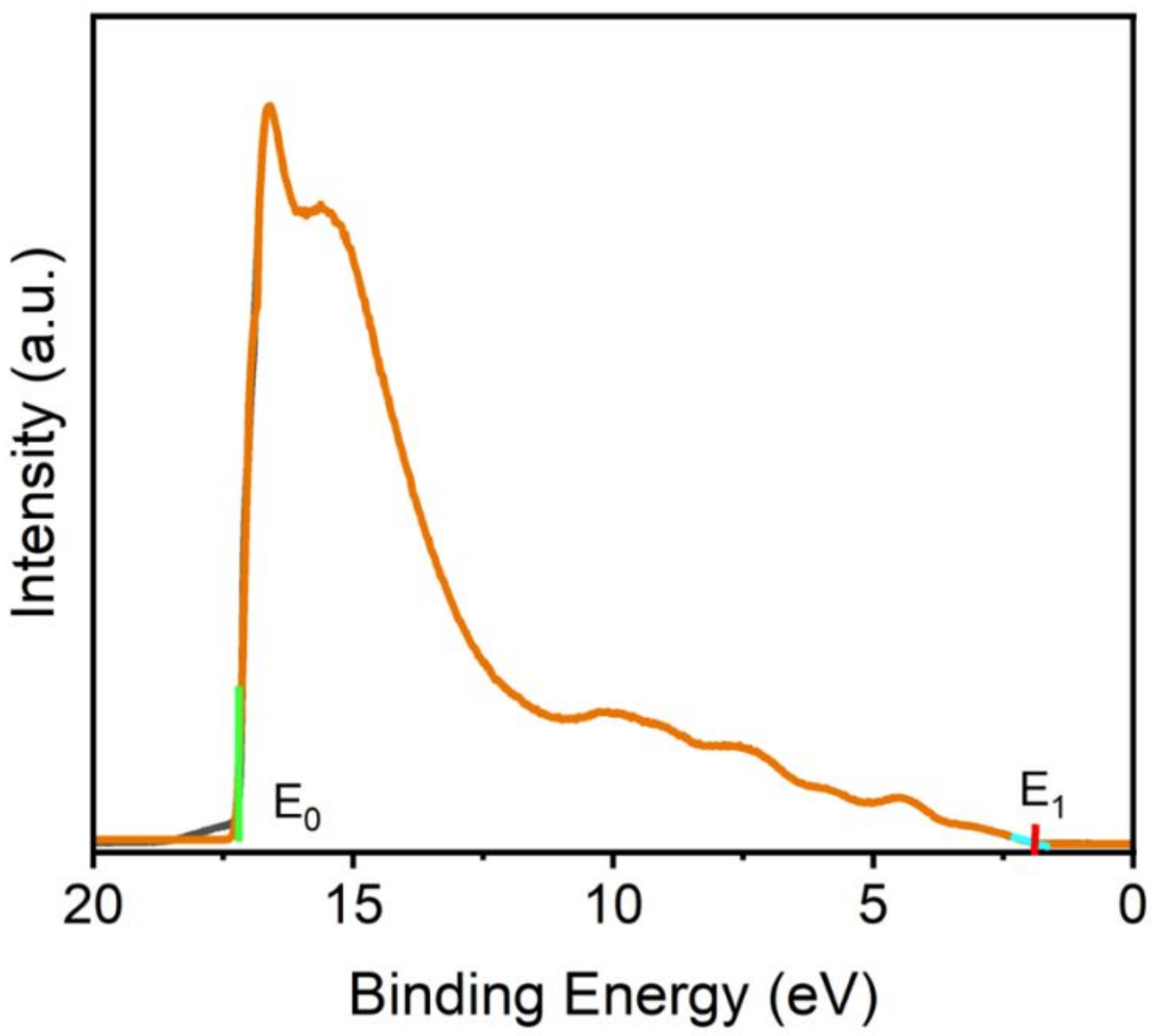

Figure S21: UPS spectrum of the TPA_2PbBr_4 perovskite thin film.

Table S8: UPS spectra parameters.

Sample	Work Function (WF)	E_{ion}	$E_1 = E_{ion} - WF$
TPA_2PbBr_4	3.99	5.90	1.91

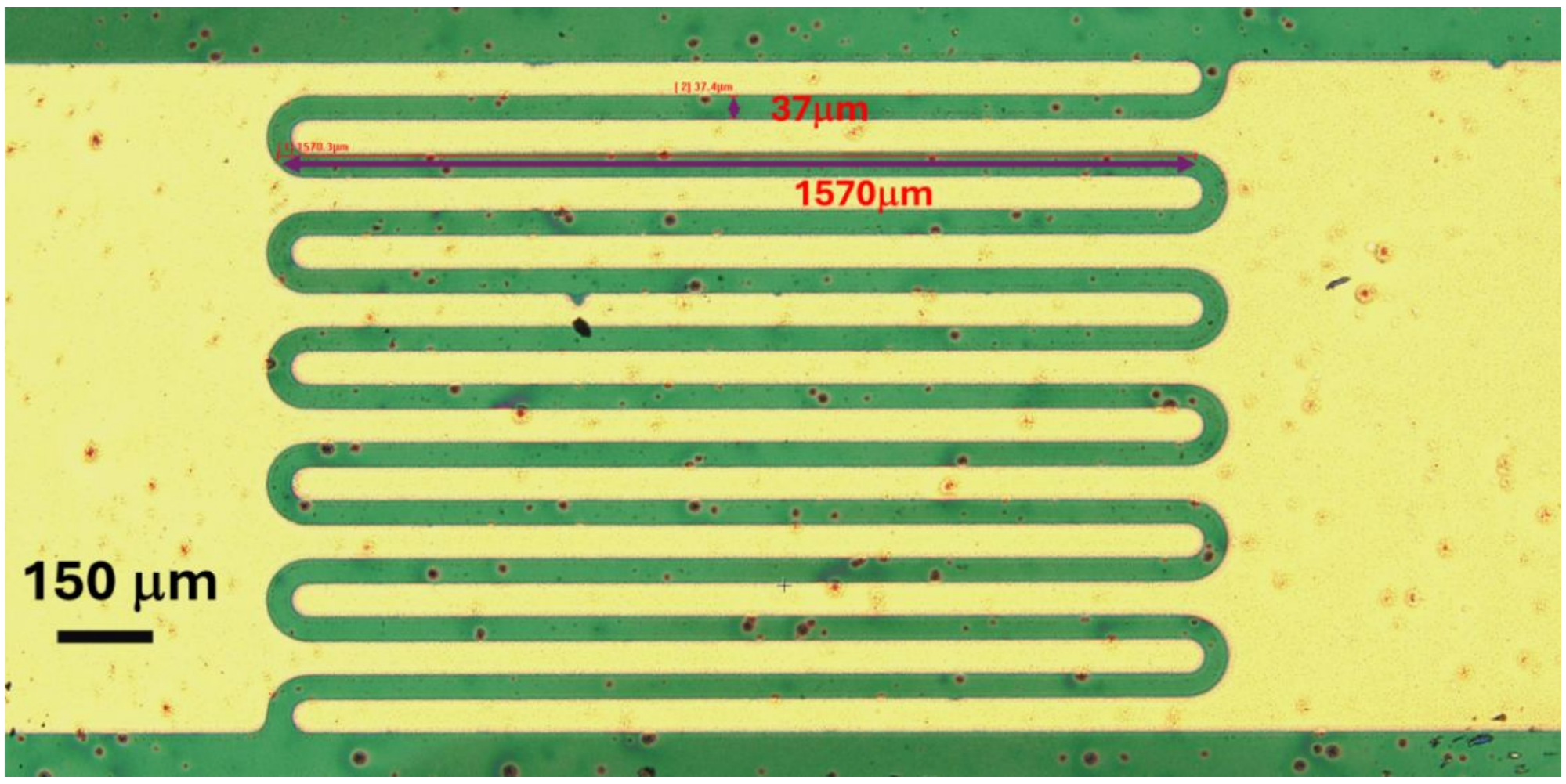

Figure S22: Optical image of the lateral TPA_2PbBr_4 perovskite PD. Length of the channel 1570 µm, and width of the channel = 37 µm × 11. The area (A) of the device is 6.4×10^{-3} cm^2.

CS1. Calculation of the lateral device performance parameters, including photoresponsivity (R), and detectivity (D*), for the Si_SiO_2/TPA_2PbBr_4/Au-based photodetector (PD) device.

The parameters of the Si/SiO_2/TPA_2PbBr_4/Au-based PD were calculated under 385 nm LED illumination with a power density of 0.03 mW.cm^{-2} and an applied bias voltage of 1 V.

The photoresponsivity (R) for the lateral TPA_2PbBr_4-based PD was estimated by using Eq. (1):

$$\mathrm{R} = \frac{\Delta I}{P(\mathrm{A})} \qquad (1)$$

where ΔI is the photocurrent, A is the active device area, and P is the illumination power (given by (LED power/LED spot area, falling on the device). Thus:

R = (2.02 × 10^{-10} A)/ (0.03 mW.cm^{-2} × 0.0064 cm^2) = 1.03 × 10^{-3} A W^{-1}.

The detectivity (D*) for the TPA_2PbBr_4-based PD was estimated by using Eq. (2)**:**

$$\mathrm{D}^* = \frac{\mathrm{R}\sqrt{A}}{\sqrt{2eI_D}} \qquad (2)$$

where I_D is the dark current (4.5 × 10^{-11} A). Thus:

D* = (1.03 × 10^{-3} A W^{-1}) × (0.0064)$^{1/2}$ / (2 × 1.6 × 10^{-19} J × 4.5 × 10^{-11} A)$^{1/2}$ = 2.17 × 10^{10} Jones.

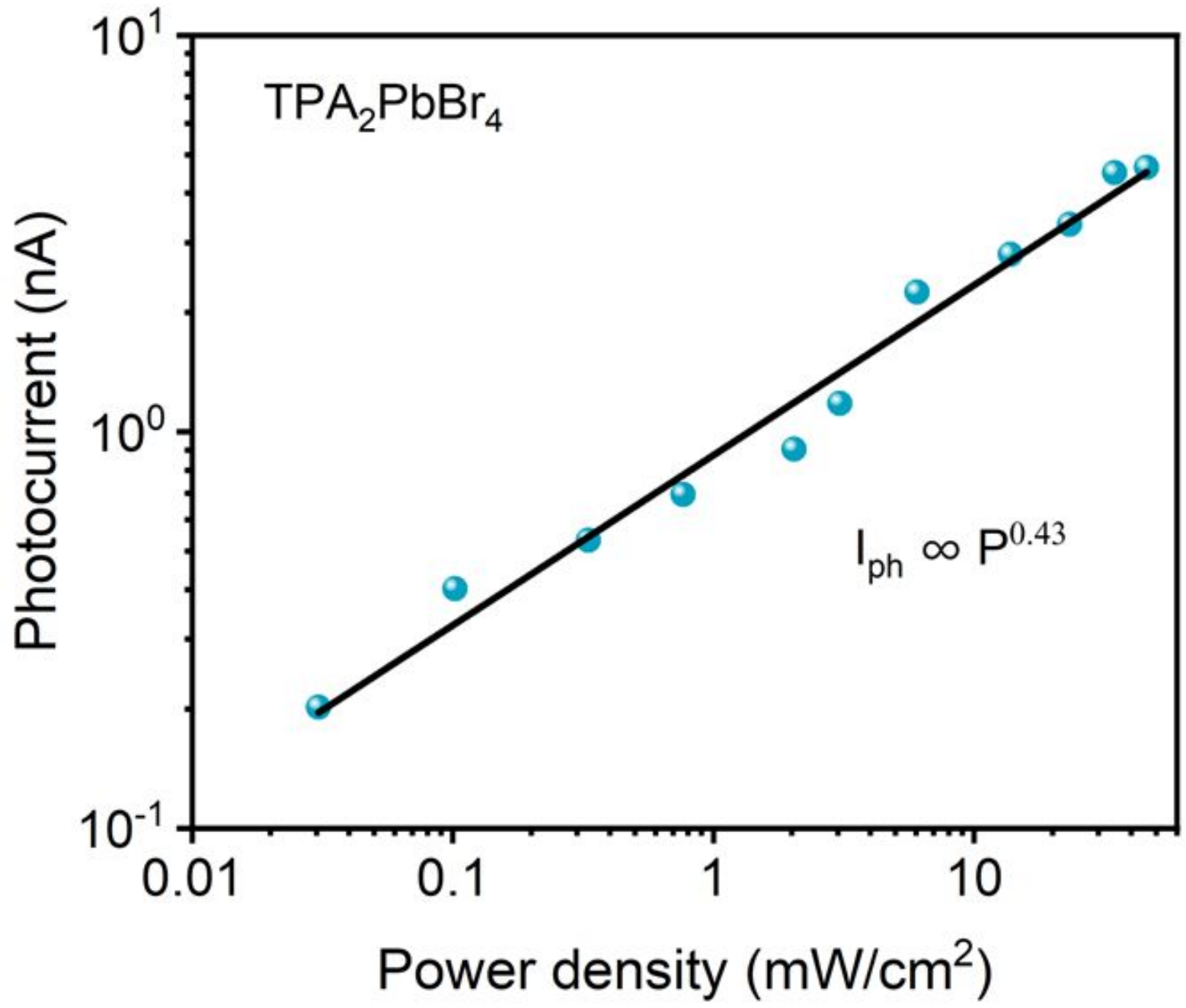

Figure S23: Log-log plot of optical power density versus photocurrent under 385 nm excitation at a fixed bias voltage of 1 V of the TPA_2PbBr_4-based planar device.

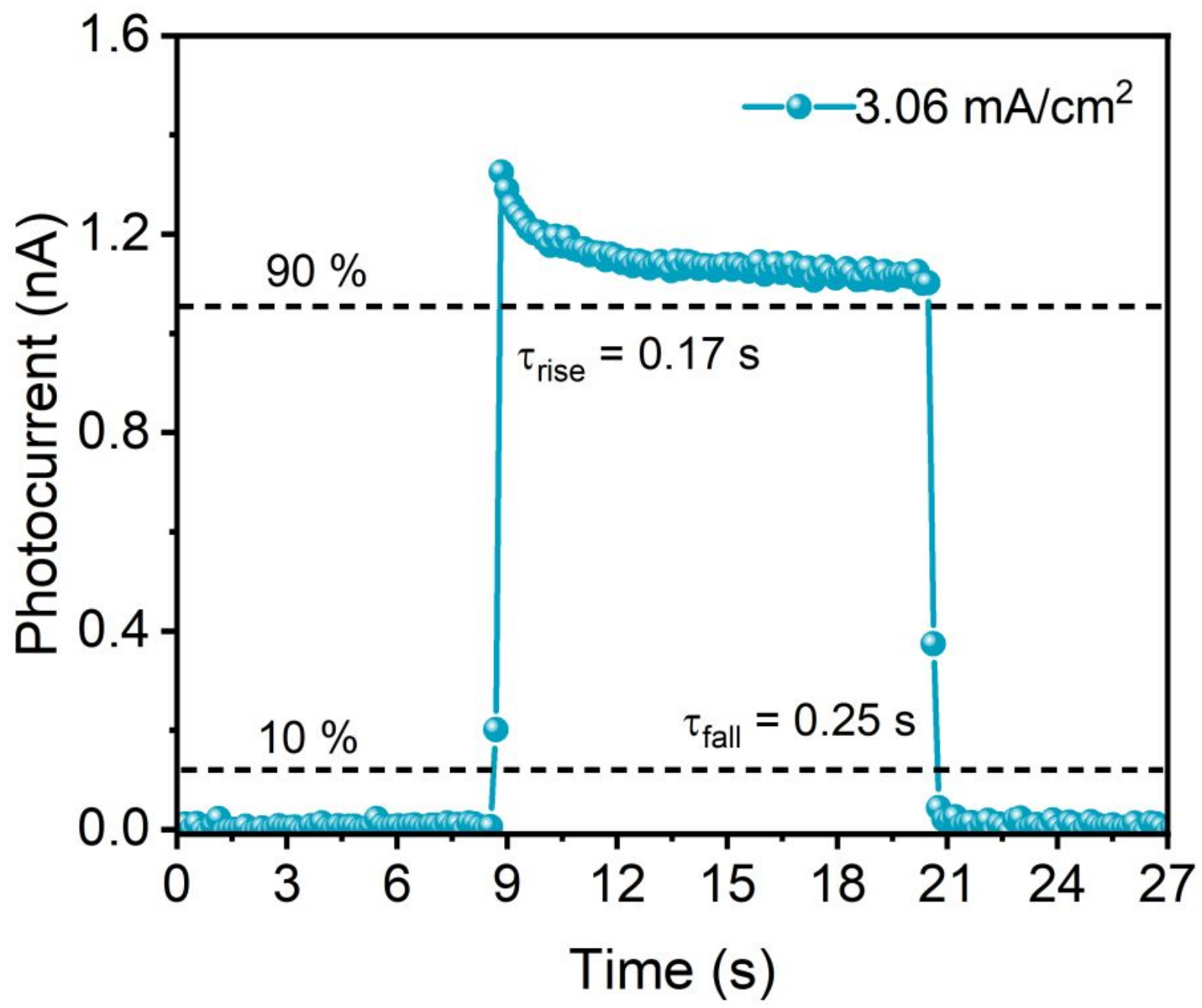

Figure S24: The response time of the TPA_2PbBr_4-based planar device was evaluated using the 90%-10% method from a single cycle of the dynamic photoresponse curve under 385 nm excitation.

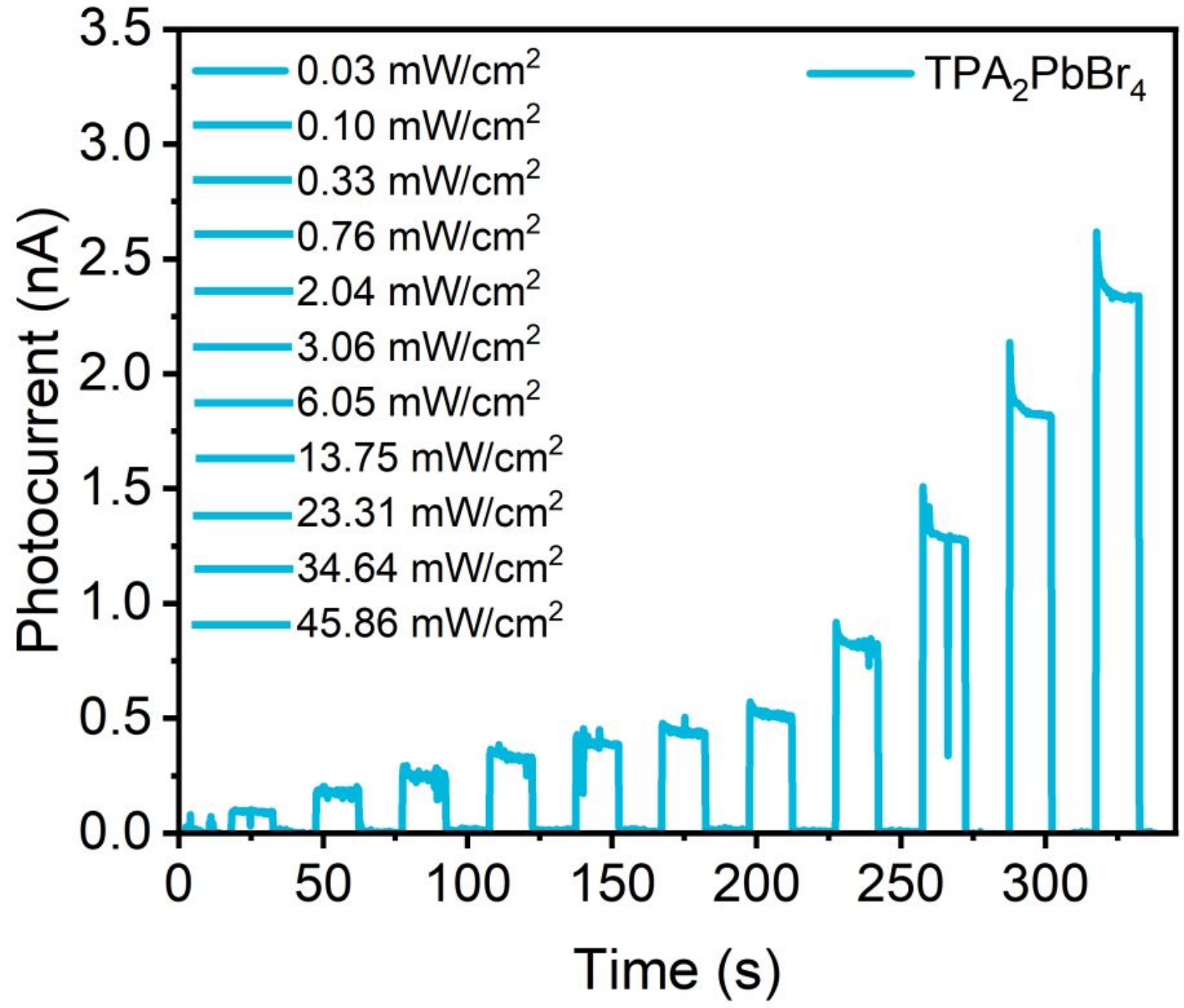

Figure S25: Stability after 3.5 months of photoresponse of the TPA_2PbBr_4 planar device under 385 nm LED with different power intensities.

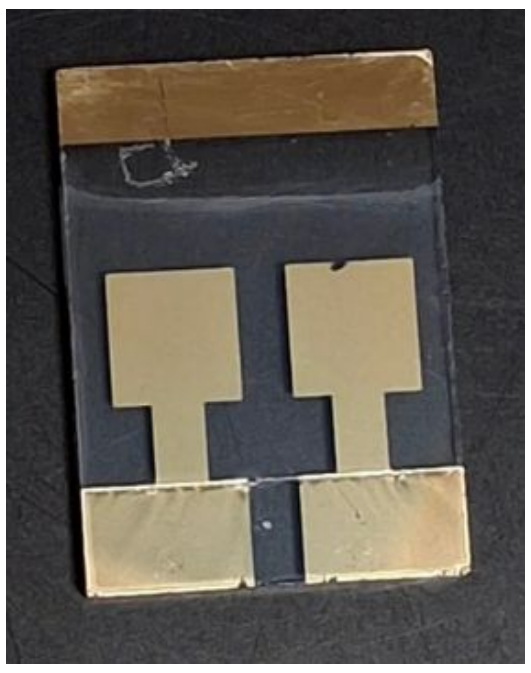

Figure S26: Photographic image of the TPA_2PbBr_4 perovskite vertical PD. Length of the active area 0.4 cm, and width of the active area = 0.4 cm. The device's area (A) is 0.16 cm^2.

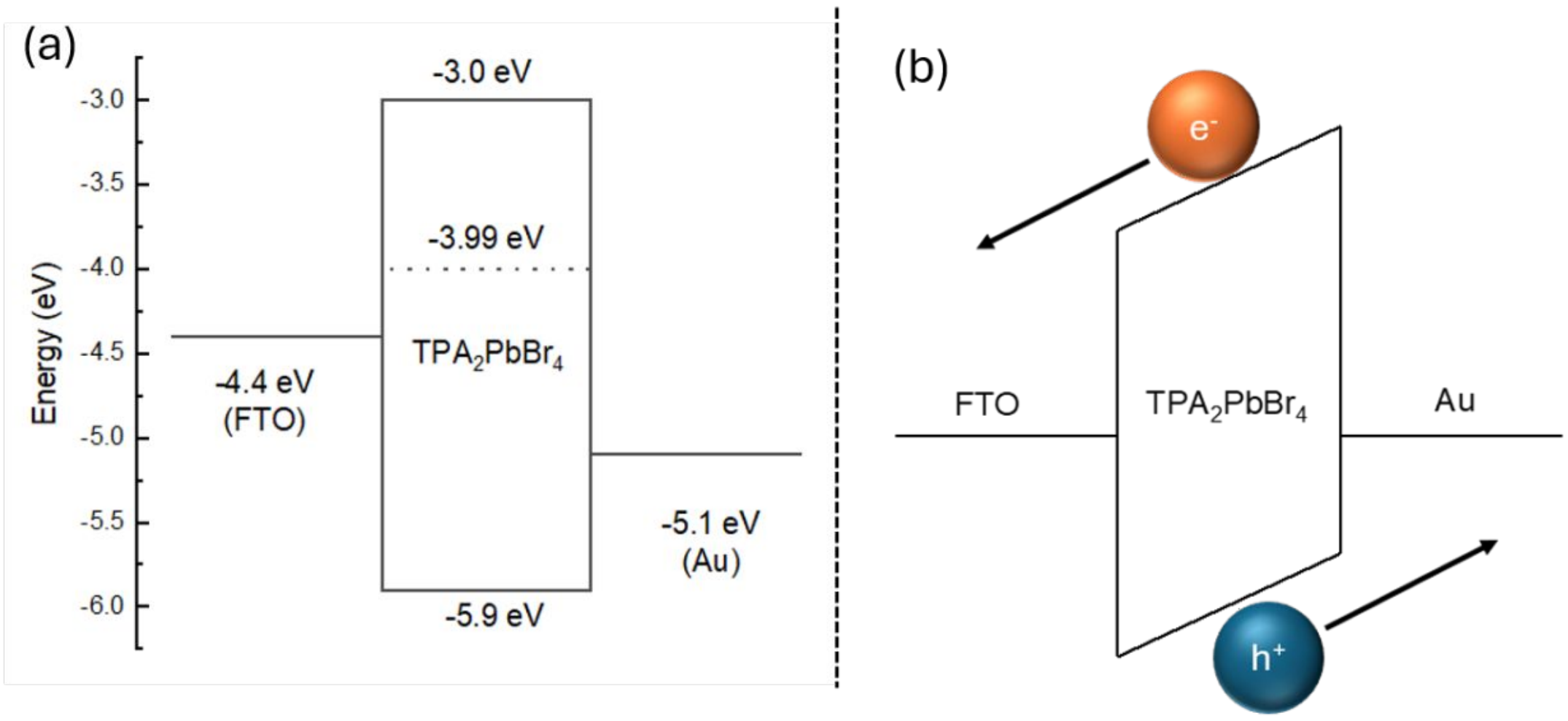

Figure S27: (a) Schematic energy band alignment of the FTO/TPA_2PbBr_4/Au vertical photodetector. (b) Illustration of the self-powered operating mechanism. The built-in electric field arising from the work function difference between the Au and FTO electrodes drives photogenerated electrons toward the FTO electrode and holes toward the Au electrode under 0 V bias conditions, enabling efficient charge separation and photocurrent generation without an external bias.

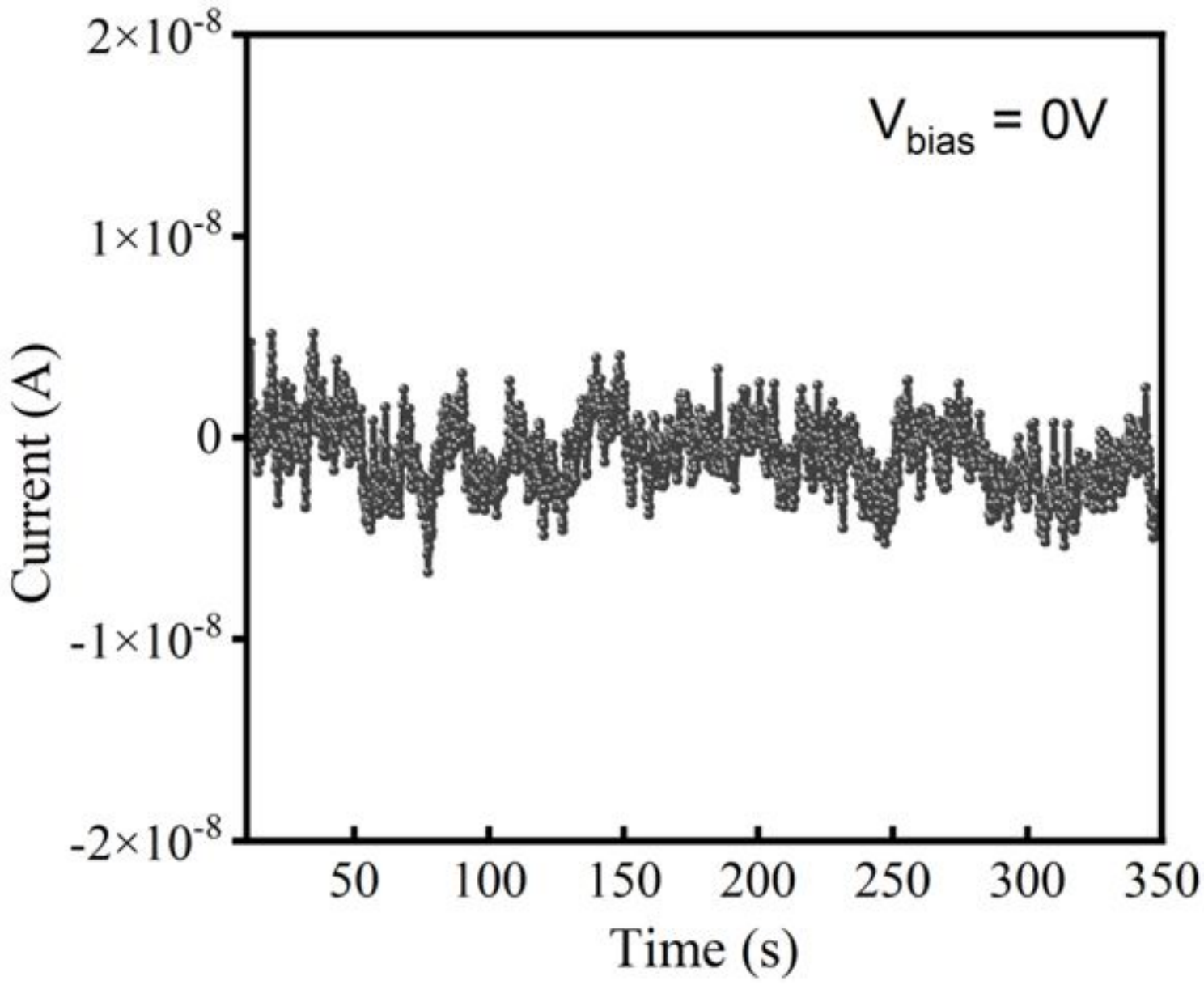

Figure S28. Dark current of the TPA2PbBr4 vertical PD at 0 V.

CS2. Calculation of the vertical device performance parameters, including photoresponsivity (R), and detectivity (D*), for the FTO/TPA_2PbBr_4/Au-based photodetector (PD) device.

The parameters of the FTO/TPA_2PbBr_4/Au-based PD were calculated under 385 nm LED illumination with a power density of 0.03 $mW.cm^{-2}$ upon self-bias operation (0 V).

The photoresponsivity (R) for the lateral TPA_2PbBr_4-based PD was estimated by using Eq. (1):

$$R = \frac{\Delta I}{P(\mathrm{A})} \qquad (1)$$

where ΔI is the photocurrent, A is the active device area, and P is the illumination power (given by (LED power/LED spot area). Thus:

$R = (3.18 \times 10^{-9}\ \mathrm{A}) / (0.03\ \mathrm{mW.cm^{-2}} \times 0.16\ \mathrm{cm^2}) = 6.5 \times 10^{-4}\ \mathrm{A\ W^{-1}}$

The detectivity (D*) for the TPA_2PbBr_4-based vertical PD was estimated by using Eq. (2)**:**

$$D^* = \frac{R\sqrt{A}}{\sqrt{2eI_D}} \qquad (2)$$

where I_D, the dark current at 0V is 5.5×10^{-10} A. Thus:

$D^* = (6.5 \times 10^{-4}\ \mathrm{A\ W^{-1}}) \times (0.16)^{1/2} / (2 \times 1.6 \times 10^{-19}\ \mathrm{J} \times 5.5 \times 10^{-10}\ \mathrm{A})^{1/2} = 2.48 \times 10^{10}$ Jones.

Table S9: Device parameters of various 2D layered perovskite-based photodetector:

Material	Peovskite Morphology	Responsivity (A/W)	Detectivity	References
BA_2PbI_4	Single Crystal	1.53	2.17 x 10^{11}	*J. Mater. Chem. C 2021,* ***9****, 11085*
BA_2PbBr_4/Graphene	Single Crystal	2.1 x 10^3	-	*J. Am. Chem. Soc. 2016,138, 16612.*
PEA_2PbI_4	Single crystal	5 x 10^{-3}	1.0 x 10^{13}	*ACS Nano 2019, 13, 1168*
PEA_2PbBr_4	Single crystal	0.0315	1.5 x 10^{13}	*J Mater Chem C. 2019; 7(6): 1584-1591*
$(BA)_2(MA)_3Pb_4I_{13}$	Single Crystal	74 x 10^3	1.2 x 10^{15}	*Nat. Mater. 2018, 17, 908−914*
PEA_2SnI_4	-	0.121	8.9 x 10^9	*ACS Appl. Mater. Interfaces 2019, 11, 8419−8427*
Cs_3BiI_9	Single Crystal	0.033	1 x 10^{10}	*Nano Res. 2019, 12, 1894*
$BA_2(MA)_{n-1}Pb_nI3_{n+1}$	Thin Film	0.12	-	*ACS Appl. Mater. Interfaces 2018, 10, 19019*
BPA_2PbBr_4	Single Crystal	1 x 10^{-4}	1 x 10^7	*Angew. Chem. 2020, 132, 19095*
$(C_{12}H_{27}N)_2(MA)n_{-1}(Pb)n(Br)_{3n+1}$	Nanostripes	0.183	2.9 x 10^{11}	*Adv. Funct. Mater.2025, 35, 2416027*
TPA_2PbBr_4	Thin Film	1 x 10^{-3}	2.2 x 10^{10}	*This work*

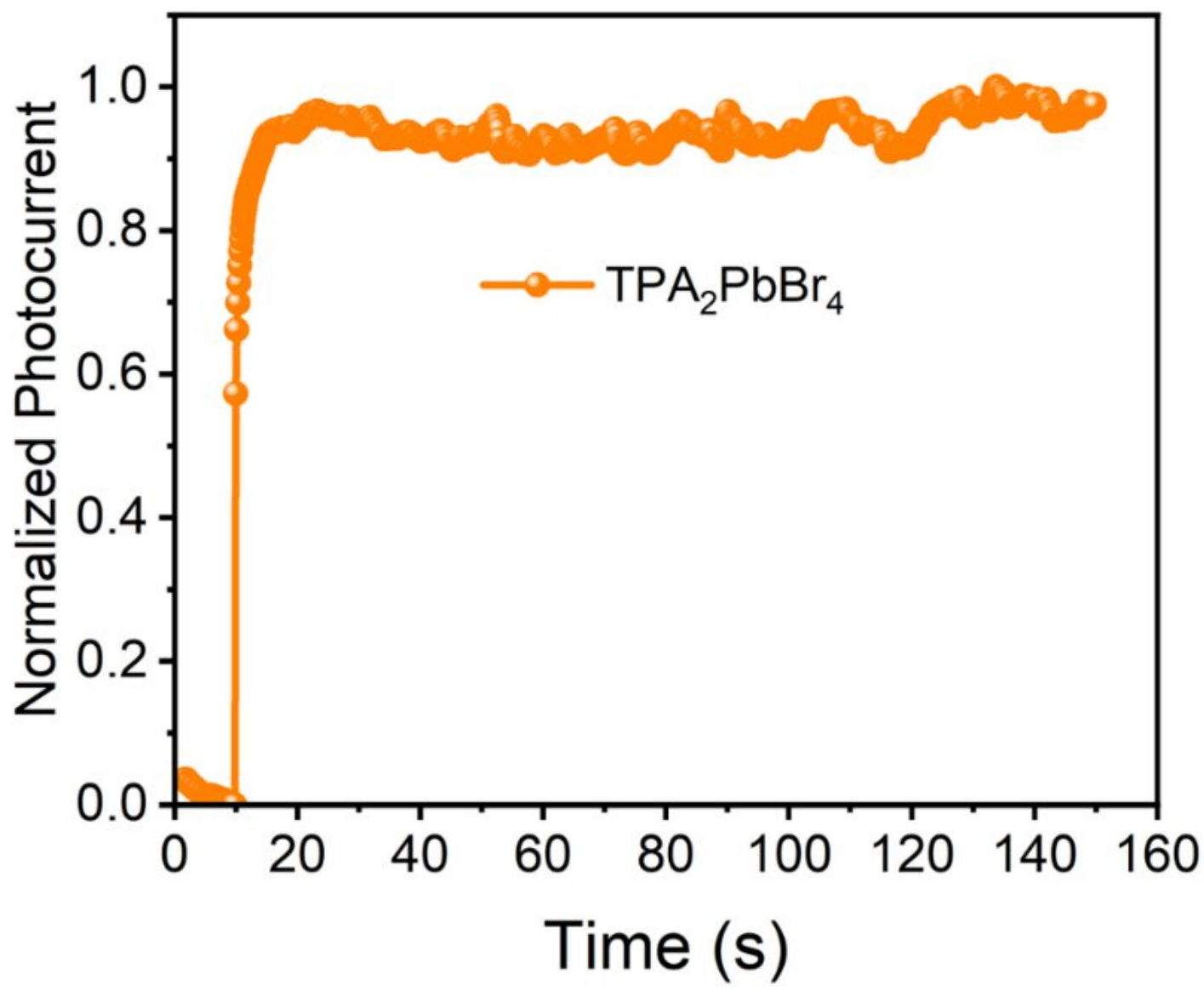

Figure S29: Stability measurement of the vertical TPA_2PbBr_4 PD upon illumination under 385 nm LED measured at 0 V bias.

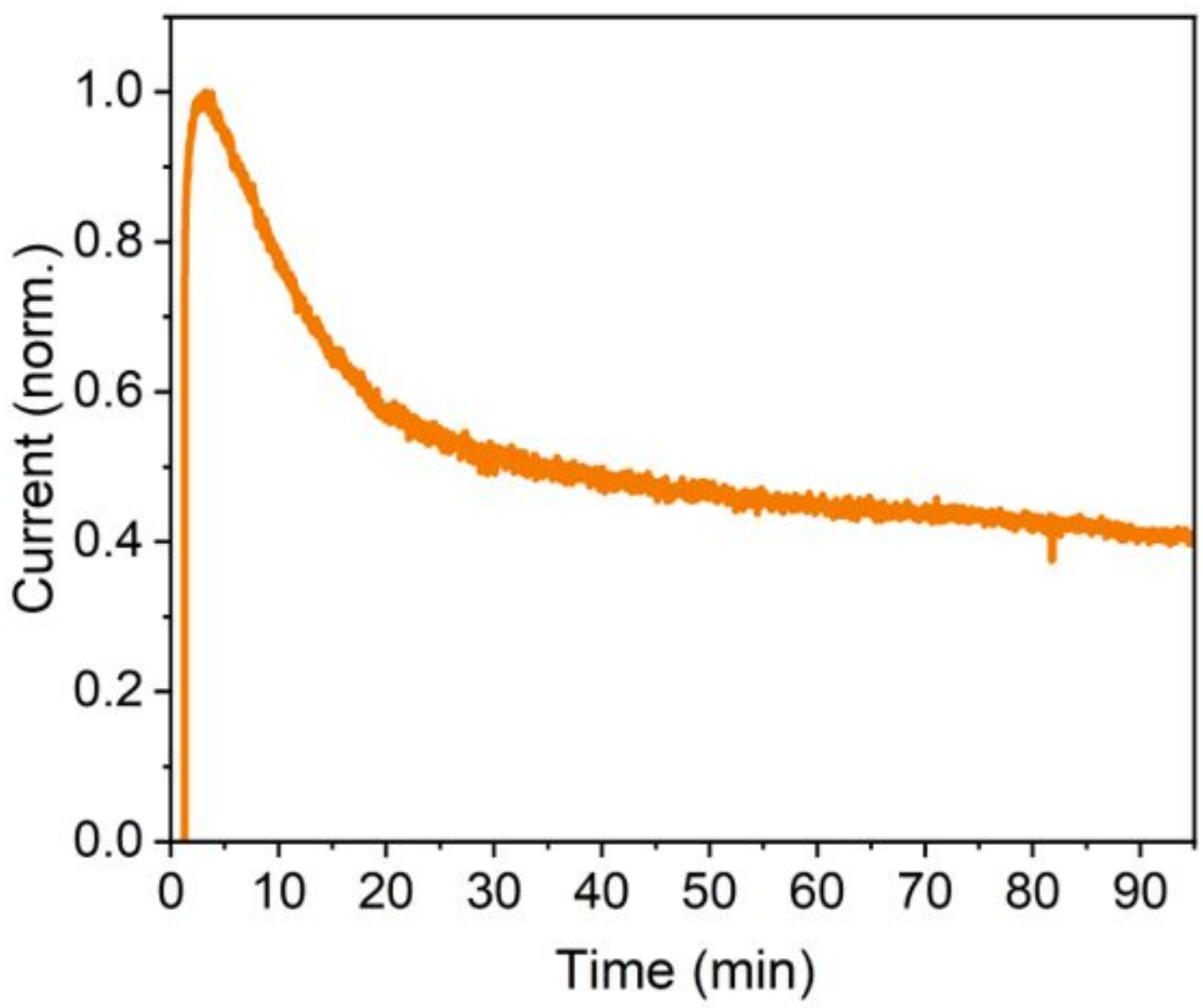

Figure S30: Operational stability of the vertical TPA_2PbBr_4 photodetector measured under continuous illumination with a 385 nm LED at 0 V bias. The photocurrent shows an initial decrease during the first ~20 min of UV exposure, followed by a stable response over the remainder of the measurement.

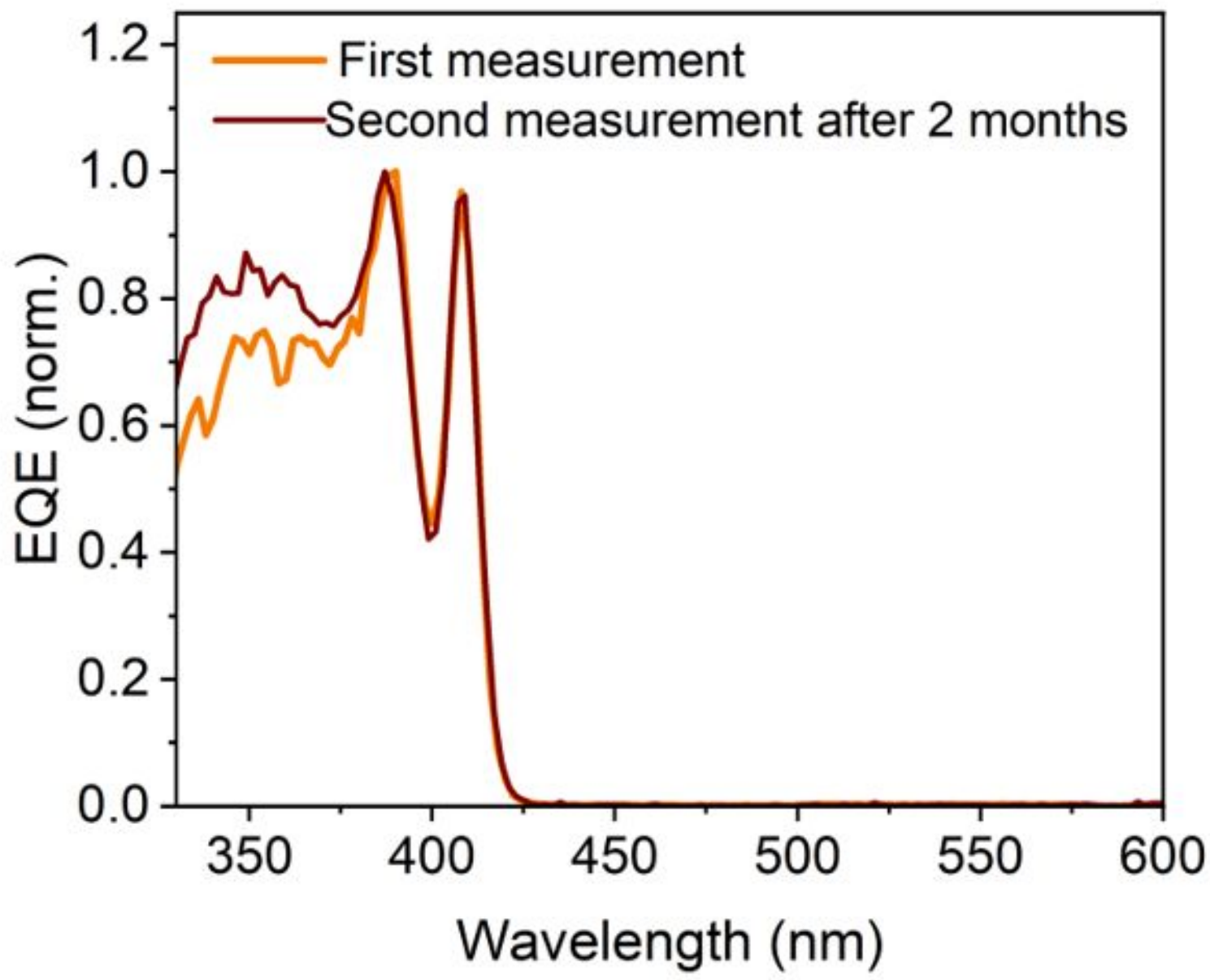

Figure S31: Normalized external quantum efficiency (EQE) spectra of the same device measured immediately after fabrication and after approximately 2 months of storage in a dry box.
